# Factors influencing adherence in a trial of early introduction of allergenic food

**DOI:** 10.1016/j.jaci.2019.06.046

**Published:** 2019-12

**Authors:** Michael R. Perkin, Henry T. Bahnson, Kirsty Logan, Tom Marrs, Suzana Radulovic, Rebecca Knibb, Joanna Craven, Carsten Flohr, E.N. Mills, Serge A. Versteeg, Ronald van Ree, Gideon Lack, Enquiring About Tolerance (EAT) study team, Louise Young, Victoria Offord, Mary DeSousa, Jason Cullen, Katherine Taylor, Anna Tseng, Bunmi Raji, Sarah Byrom, Gillian Regis, Charlie Bigwood, Charlotte Stedman, Sharon Tonner, Emily Banks, Yasmin Kahnum, Rachel Babic, Ben Stockwell, Erin Thompson, Lorna Wheatley, Devi Patkunam

**Affiliations:** a Population Health Research Institute, St George's, University of London, London, United Kingdom; b Benaroya Research Institute, Seattle, Wash; c Paediatric Allergy Research Group Department of Women and Children's Health, School of Life Course Sciences, King's College London, London, United Kingdom; d Department of Psychology, Aston University, Birmingham, United Kingdom; e Unit for Population-Based Dermatology Research, St John's Institute of Dermatology, School of Basic and Medical Biosciences, Faculty of Life Sciences & Medicine, King's College London, London, United Kingdom; f School of Biological Sciences, Division of Infection, Immunity and Respiratory Medicine, Manchester Academic Health Science Centre, and the Manchester Institute of Biotechnology, University of Manchester, Manchester, United Kingdom; g Department of Experimental Immunology, Academic Medical Center, Amsterdam, the Netherlands; h Department of Otorhinolaryngology, Academic Medical Center, Amsterdam, the Netherlands

**Keywords:** Food allergy, diet, allergens, infancy, breastfeeding, randomized controlled trial, adherence, EAT, Enquiring About Tolerance, EIG, Early introduction group, LEAP, Learning Early About Peanut Allergy, SIG, Standard introduction group

## Abstract

**Background:**

The Enquiring About Tolerance (EAT) study examined whether the early introduction of 6 allergenic foods from 3 months of age in exclusively breastfed infants prevented the development of food allergy. The intervention was effective in the per-protocol analysis for allergy to 1 or more foods and for egg and peanut individually, but only 42% of early introduction group (EIG) children met the per-protocol criteria.

**Objective:**

We sought to identify which factors were responsible for nonadherence in the EAT study.

**Methods:**

Factors influencing adherence within the key early introduction period in the EIG (up to 6 months of age) were divided into enrollment and postenrollment factors, and their association with nonadherence was explored.

**Results:**

In an adjusted analysis, at enrollment, increased maternal age, nonwhite ethnicity, and lower maternal quality of life were independently and significantly associated with overall nonadherence in the EIG. Enrollment eczema and enrollment serum allergen-specific IgE sensitization to 1 or more foods (≥0.1 kU/L) were not related to overall nonadherence. After enrollment, 2 factors were significantly related to EIG overall nonadherence: parent-reported IgE-type symptoms with infant allergenic food consumption by 6 months of age and reported feeding difficulties by 4 months of age.

**Conclusion:**

If early introduction of allergenic foods were to be considered a strategy to prevent food allergy, families of nonwhite ethnicity, those with older mothers, and those with infants with reported feeding difficulties or early-onset eczema would benefit from support to promote early and sustained consumption.

## Graphical abstract

**Figure:**
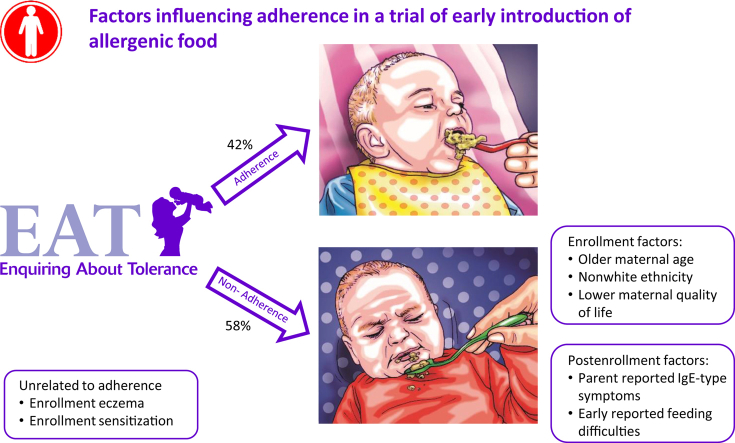

The Enquiring About Tolerance (EAT) study was a large randomized food allergy prevention trial of the early introduction of 6 allergenic foods from 3 months of age in exclusively breast-fed infants recruited from the general population.^1^, ^2^ The EAT study achieved markedly different rates of exposure to allergenic foods between the randomized groups (the early introduction group [EIG] and the standard introduction group [SIG]) before 6 months of age.^1^ However, the study did not show statistically significant efficacy in an intention-to-treat analysis but did show a significant per-protocol effect for allergy to 1 or more foods and for egg and peanut individually.^2^

There are 2 explanations for this discrepancy. First, the per-protocol analysis was effective because the population adhered to the intervention. Second, the intention-to-treat analysis was not effective because among the large group of children who did not adhere to treatment, those with nascent allergy with food sensitization had subclinical symptoms, food aversion, or both and were therefore unable to adhere. Further analyses suggested that food allergy prevention through early introduction of multiple allergenic foods in normal breastfed infants might depend on adherence and dosage.^2^

The EAT study per-protocol criteria for the EIG were stringent. By 6 months of age, EIG infants were expected to have achieved sustained high-dose consumption of 5 or more of the 6 early introduction foods. Achieving such per-protocol adherence in the EIG proved difficult. Only 42% (223/529) of adherence-evaluable EIG children complied entirely with the protocol (34% (223/652) of the whole EIG group).

After the completion of the study, we measured serum allergen-specific IgE sensitization in the EAT participants and demonstrated in an intention-to-treat analysis that the intervention successfully reduced the development of food allergy among EIG infants who were sensitized on specific IgE testing at enrollment. Infants with the early emergence of food-specific IgE sensitization to foods are known to be at high risk of developing a food allergy.^3^, ^4^

In this article we explore how these and other data collected in the EAT study influenced the issue of nonadherence to the recommended level of consumption of the 6 early introduction foods (overall nonadherence), as well as nonadherence to consumption of individual foods (food-specific nonadherence). We determined whether occult sensitization and early symptoms were responsible for the high nonadherence rate.

The UK Scientific Advisory Committee on Nutrition has issued the following statement after its review of the evidence on feeding in the first year of life: “The available evidence indicates that the deliberate exclusion or delayed introduction of peanut or hen's egg beyond 6 to 12 months of age may increase the risk of allergy to the same foods.”^5^ We address how barriers to the prompt introduction of allergenic foods might be mitigated, thereby enhancing the possibility of food allergy prevention.

## Methods

### Participants

One thousand three hundred three 3-month-old infants were recruited from the general population in England and Wales through direct advertising and were enrolled between November 2009 and July 2012. Details of the EAT study methodology have been published elsewhere.^1^ Maternal age at enrollment ranged from 19 to 46 years (median, 33 years), and there was no difference in maternal age between study groups. The median age was used as the cutoff for dividing mothers into younger (<33 years) and older (≥33 years) groups. All children were generally well, exclusively breastfed, and born at term (≥37 weeks' gestation). Ethnic origin of the child was based on their parent-defined ethnicity coded by using the classification used in the 2001 UK Census.^6^ One thousand one hundred four (84.7%) of the enrolled infants were white, 119 (9.1%) were mixed, and 80 (6.1%) were black, Asian, or Chinese. The trial was registered with the ISRCTN (registration no. 14254740). Ethical approval for the EAT study was provided by St Thomas' Hospital REC (REC reference 08/H0802/93), and informed consent was obtained from the parents of all children enrolled in the study.

### Procedures

Participants were randomized to the SIG or the EIG. The SIG was asked to exclusively breastfeed to around 6 months of age in accordance with the recommendation in place in the United Kingdom since 2003.^7^ Beyond 6 months, allergenic food consumption was at parental discretion.

The EIG parents were asked to introduce 6 allergenic foods to their infants: cow's milk (yogurt), peanut, cooked (boiled) hen's egg, sesame, white fish, and wheat.^1^ Cow's milk was always introduced first as yogurt. The order of introduction of the next 4 foods was randomly determined to avoid any bias from one food being introduced consistently before another. Wheat was introduced last and not before 4 months of age (see the “EIG early introduction regimen” section in the Methods section in this article's Online Repository at www.jacionline.org).

An online questionnaire was sent to each family monthly to 1 year of age and then every 3 months until the child reached 3 years of age. Each questionnaire allowed families to report any suspected symptoms with food ingestion and to list suspected foods. Clinical judgement was used to divide these into IgE-type and non–IgE-type symptoms. The online questionnaires also ascertained maternal quality of life using the World Health Organization Quality of Life BREF questionnaire,^8^ infant sleep using the Brief Infant Sleep Questionnaire,^9^ and parental reporting of feeding difficulty and aversive feeding behavior by using questions based on previously published work.^10^ Clinic visits took place at enrollment and at 1 and 3 years of age. Further information on the online questionnaire and clinic visit data collected are presented in the “Information collected” section in the Methods section in this article's Online Repository.

Food-specific IgE levels to each of the 6 foods were measured at enrollment and at 1 and 3 years of age in both groups using ImmunoCAP (Phadia, Uppsala, Sweden) assays. The primary outcome of the EAT study was the proportion of participants with challenge-proved food allergy to 1 or more of the 6 early introduction foods between 1 and 3 years of age.^2^

### Per-protocol adherence

The definitions of overall per-protocol adherence are presented in Table E1 in this article's Online Repository at www.jacionline.org. The key criterion for overall adherence in the EIG was consumption of at least 5 of the allergenic foods in at least 75% of the recommended amount (3 g of allergen protein/wk) for at least 5 weeks between 3 and 6 months of age. Food-specific per-protocol adherence was based on the same criteria (ie, consumption of ≥75% of the recommended amount of the specific food for at least 5 weeks between 3 and 6 months of age). The window in which 5 or more weeks of consumption could be achieved was narrow, as explained in more detail in the “Restricted window to achieve per-protocol status” section in the Methods section in this article's Online Repository.

### Statistical analyses

Univariate and multivariable logistic regression models were used to identify factors associated with nonadherence. Penalized logistic regression was used where appropriate for situations with very sparse data. Eczema was analyzed as the presence of visible eczema at enrollment by using the UK diagnostic criteria (“visible eczema”).^11^ Eczema severity was determined by using the SCORAD index and grouped by using the accepted SCORAD categories.^12^ Data were analyzed with STATA software (version 15; StataCorp, College Station, Tex), SAS software (version 9.4; SAS Institute, Cary, NC), and JMP Pro 14 (SAS Institute). The EAT data set (ITN900AD) is available through TrialShare, a public Web site managed by the Immune Tolerance Network (www.itntrialshare.org).

## Results

Overall per-protocol adherence status could only be determined in 81% (529/652) of the EIG participants (see Table E1). An exploration of the likely true adherence status of the 123 EIG participants (19% of the EIG) whose adherence status was nonevaluable, as well as the effect of including or excluding them in the analyses undertaken for this article, are reviewed in detail in the “EIG adherence nonevaluable participants” section in the Results section and in Table E2 and Figs E1 and E2 in this article's Online Repository at www.jacionline.org.

**Fig 1:**
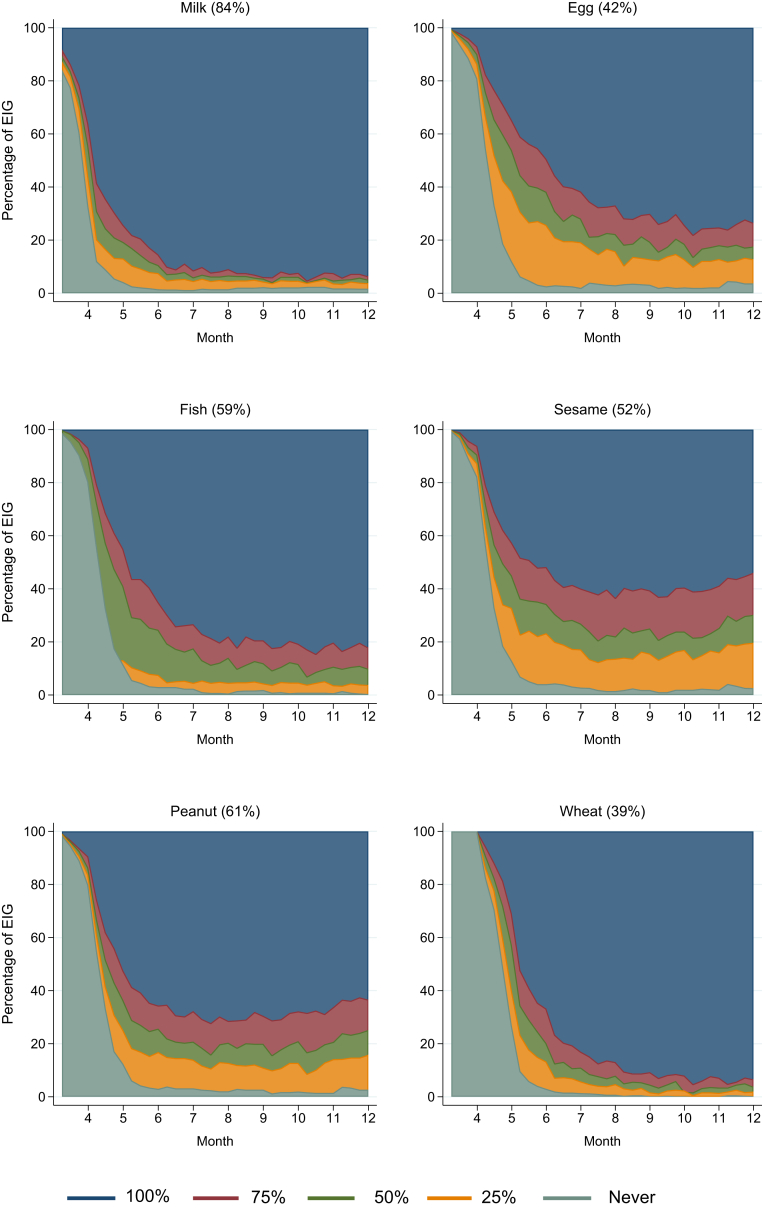
Adherence with introduction of allergenic foods in the EIG up to 1 year of age. The figure presents the relative proportions of the EIG consuming 100%, 75% (the per-protocol threshold), 50%, or 25% or not having started consuming each of the 6 early introduction foods from enrollment through 12 months of age. The food-specific per-protocol adherence percentage (among those whose food-specific adherence status was evaluable) is shown in parentheses.

Although overall adherence to the EAT early introduction regimen was low at 42%, families found it easier to introduce some foods than others (Fig 1), with the result being that food-specific adherence varied by food: milk, 84% (451/537); peanut, 61% (336/549); whitefish, 59% (318/543); sesame, 52% (288/550); egg, 42% (234/551); and wheat, 39% (216/553, see Table E3 in this article's Online Repository at www.jacionline.org). Although randomization had been effective in ensuring the median age of introduction of peanut, egg, sesame, and fish was the same at 19.6 weeks, there remained the possibility that the position in the order in which a specific food was introduced might be associated with overall or food-specific adherence. Specific foods introduced later in the sequence reduced the likelihood of being per-protocol adherent to that food (see Fig E3 in this article's Online Repository at www.jacionline.org). However, the order of introduction of each of the foods was unrelated to overall per-protocol adherence.

The key period for defining per-protocol adherence was consumption through 6 months of age. However, Fig 1 clearly indicates that there were certain foods in which the ability to meet the per-protocol threshold (3 g/wk of allergen protein) continued to improve between 6 and 12 months of age. High sustained levels of consumption were achieved by 6 months of age for milk and shortly after 6 months of age for wheat. Per-protocol consumption of fish and egg continued to improve beyond 6 months, plateauing at 9 months for fish and 10 months for egg. Most remarkable were the patterns observed for sesame and peanut. The level of consumption of these 2 foods that had been achieved by 6 months of age did not materially change throughout the rest of the first year of life. Hence by 1 year of age, there was still a significant minority who were not eating peanut or sesame at the per-protocol recommended level.

### Enrollment factors associated with EIG overall and food-specific nonadherence (univariate analysis)

Nonwhite ethnicity, increased maternal age, and lower enrollment maternal quality-of-life scores were significantly associated with not being per-protocol adherent in the EIG (see Table E4 in this article's Online Repository at www.jacionline.org for univariate analysis). Nonwhite ethnicity and increased maternal age were associated with delaying the introduction of solids in both the SIG and EIG infants (see Fig E4 in this article's Online Repository at www.jacionline.org). Differences were observed in other enrollment demographic characteristics between ethnic groups: visible eczema and enrollment sensitization were both more common in nonwhite ethnic groups, and a history of a sibling having a parent-reported food allergy was more common in infants of Asian, black, or Chinese ethnicity (see Table E5 in this article's Online Repository at www.jacionline.org) and are reviewed in the “Association between ethnicity and baseline demographic characteristics” in the Results section in this article's Online Repository.

Eczema severity (SCORAD as a continuous variable) was significantly associated with overall nonadherence, whereas any visible eczema at enrollment was of borderline significance (*P* = .07, see Table E4). More details about the associations between enrollment factors and overall nonadherence are presented in the “Enrollment factors associated with overall nonadherence” section in the Results section in this article's Online Repository. Associations between enrollment factors and food-specific nonadherence are presented in the “Enrollment factors associated with food-specific nonadherence” section in the Results section in this article's Online Repository.

### Enrollment sensitization in the EIG

In the EAT study there were too few EIG participants sensitized on skin prick testing at enrollment to individual foods (ranging from none for sesame to 24 for raw egg white) to be able to determine reliably the effect on subsequent food-specific adherence (Table I). More EIG participants were shown to be sensitized to specific foods based on serum food-specific IgE testing using a 0.1 kU/L threshold (response to ≥1 foods of ≥0.1 kU/L; 15.7% [93/593]) than were identified to have positive skin prick test responses (>0 mm; 5.1% [33/652]; Table I).

**Table I:** Enrollment sensitization data from the EAT study

	Any food	Peanut	Egg	Milk	Sesame	Fish	Wheat
SPT >0 mm							
EIG	5.1% (33/652)	1.2% (8/652)	3.7% (24/652)	1.4% (9/652)	0% (0/652)	0.2% (1/652)	0.2% (1/652)
EIG per-protocol	4.0% (9/223)	0.3% (1/336)	2.6% (6/234)	0.4% (2/451)†	0% (0/288)	0% (0/318)	0% (0/216)
EIG non–per-protocol	4.0% (12/302)	1.9% (4/211)	4.4% (14/315)	4.9% (4/82)	0% (0/262)	0% (0/225)	0% (0/336)
EIG adherence nonevaluable	4.2% (5/120)	1.0% (1/103)	2.0% (2/101)	0% (0/115)	0% (0/102)	0% (0/109)	0% (0/99)
Specific IgE ≥0.1 kU/L							
All participants	15.6% (182/1170)	3.6% (42/1166)	6.7% (78/1170)	6.0% (70/1169)	2.0% (23/1151)	0% (0/1164)	4.3% (50/1165)
SIG	15.4% (89/577)	3.1% (18/576)	7.3% (42/577)	6.6% (38/576)	1.4% (8/572)	0% (0/575)	4.3% (25/576)
EIG	15.7% (93/593)	4.1% (24/590)	6.1% (36/593)	5.4% (32/593)	2.6% (15/579)	0% (0/589)	4.2% (25/589)
EIG per-protocol	13.6% (28/206)	2.3% (7/305)∗	3.3% (7/214)∗	3.9% (16/414)	2.3% (6/263)	0% (0/289)	5.5% (11/201)
EIG non–Per-protocol	15.4% (42/273)	5.7% (11/193)	8.0% (23/287)	8.3% (6/72)	3.1% (7/227)	0% (0/204)	3.7% (11/301)
EIG adherence nonevaluable	15.0% (16/107)	4.4% (4/90)	4.4% (4/90)	5.8% (6/103)	2.3% (2/89)	0% (0/96)	2.3% (2/86)
Specific IgE ≥0.35 kU/L							
All participants	6.4% (74/1170)	1.6% (19/1166)	3.9% (45/1170)	2.8% (33/1169)	0% (0/1151)	0% (0/1164)	0.9% (10/1165)
SIG	6.9% (40/577)	1.6% (9/576)	4.7% (27/577)	3.3% (19/576)	0% (0/572)	0% (0/575)	0.7% (4/576)
EIG	5.7% (34/593)	1.7% (10/590)	3.0% (18/593)	2.4% (14/593)	0% (0/579)	0% (0/589)	1.0% (6/589)
EIG per-protocol	2.9% (6/206)	0% (0/305)∗	1.4% (3/214)	0.7% (3/414)∗	0% (0/263)	0% (0/289)	0.5% (1/201)
EIG non–per-protocol	5.1% (14/273)	2.1% (4/193)	3.8% (11/287)	5.6% (4/72)	0% (0/227)	0% (0/204)	1.3% (4/301)
EIG adherence nonevaluable	6.5% (7/107)	4.4% (4/90)	2.2% (2/90)	2.9% (3/103)	0% (0/89)	0% (0/96)	0% (0/86)

Rows showing the EIG divided into the per-protocol, non–per-protocol, and adherence nonevaluable subgroups show overall adherence status for the any food column and food-specific adherence status for individual food columns. Specific IgE levels were measured in 1170 children. However, some infants had very small amounts of serum obtained, and all 6 individual foods could not be measured. Hence the denominator for individual foods varies (ranging from n = 1151 for sesame to n = 1170 for egg). The 7 EIG participants who had positive enrollment challenge results to a food are excluded from the adherence rows (4 to milk, 1 to wheat, 2 to peanut, and 2 to egg) because they were unable to be adherent being already allergic to the food.

*SPT*, Skin prick test.

∗ *P* < .05 and †*P* < .01, *P* values for the EIG per-protocol and EIG non–per-protocol groups.

Food-specific IgE sensitization at enrollment strongly predicted the development of food allergy to the same food, and sensitization to 1 or more foods strongly predicted overall food allergy (see Fig E5 in this article's Online Repository at www.jacionline.org). Sensitization to 1 food also predicted food allergy developing to other foods. The group of infants with early food-specific IgE sensitization accounted for 69% of the food allergy cases that developed in the EAT study. We have shown that the EAT study early introduction intervention was effective in an intention-to-treat analysis of this high-risk population.^13^

**Fig 2:**
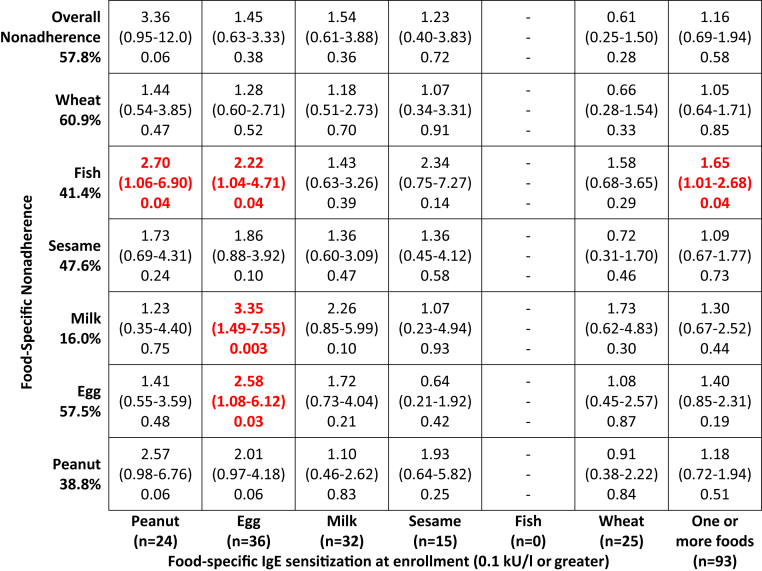
EIG enrollment IgE sensitization and overall and food-specific per-protocol adherence. Penalized logistic regression of the association between enrollment IgE sensitization (≥0.1 kU/L) to specific foods or to 1 or more of the 6 early introduction foods and the association with food-specific and overall nonadherence are shown.

The great majority of EIG infants sensitized to a specific food at enrollment did not report any symptoms when that food was introduced into their diets (eg, 92% [18/22] for peanut). Similarly, the great majority of infants whose families reported symptoms with a specific food were not sensitized to that food at enrollment (eg, 81% [17/21] for peanut). Both are reviewed in detail in the “IgE-type symptom reporting and enrollment sensitization” section in the Results section in this article's Online Repository. Serum food-specific sensitization to 1 or more foods did not predict overall nonadherence in the univariate analysis (Fig 2). In contrast, in the univariate analysis food-specific sensitization predicted food-specific nonadherence for egg and was of borderline significance for peanut (Fig 2 and see Fig E6 in this article's Online Repository at www.jacionline.org), both of which are likely to be a consequence of the study design and discussed in more detail in the “Consumption of each allergenic food by enrollment-specific IgE sensitization status” section in the Results section in this article's Online Repository.

### Enrollment factors associated with EIG overall nonadherence (multivariable analysis)

A multivariable analysis was undertaken to determine whether overall nonadherence in the EIG could have been predicted from certain enrollment characteristics (Table II, left column). There was no significant relationship between enrollment sensitization to 1 or more foods and overall nonadherence.

**Table II:** Logistic regression modelling of enrollment factors influencing EIG overall and food-specific nonadherence

	EIG overall nonadherence		EIG-specific food nonadherence											
			Peanut		Egg		Milk		Sesame		Fish		Wheat	
	OR	*P* value	OR	*P* value	OR	*P* value	OR	*P* value	OR	*P* value	OR	*P* value	OR	*P* value
Ethnicity (nonwhite)	2.19 (1.13-4.25)	.02	2.16 (1.20-3.91)	.01	1.67 (0.90-3.10)	.11	1.67 (0.79-3.50)	.18	1.68 (0.93-3.02)	.08	2.06 (1.14-3.73)	.02	1.68 (0.90-3.14)	.10
Visible eczema at enrollment (continuous SCORAD score)	1.02 (0.99-1.06)	.16	1.02 (0.99-1.05)	.21	1.02 (0.99-1.05)	.27	1.01 (0.98-1.05)	.41	1.04 (1.01-1.07)	.02	1.04 (1.01-1.07)	.01	1.01 (0.98-1.04)	.43
QOL psychological domain (< mean)	1.51 (1.02-2.22)	.04	1.17 (0.79-1.72)	.44	1.21 (0.82-1.77)	.34	0.99 (0.57-1.69)	.96	1.06 (0.72-1.54)	.77	1.02 (0.70-1.50)	.91	1.42 (0.97-2.07)	.07
Food-specific IgE at enrollment∗ (≥0.1 kU/L)	0.88 (0.48-1.60)	.68	1.18 (0.39-3.59)	.77	2.32 (0.85-6.31)	.10	1.33 (0.39-4.56)	.65	0.94 (0.28-3.14)	.92	—	—	0.47 (0.18-1.24)	.13
Maternal age (≥ median, 33 y)	1.59 (1.08-2.33)	.02	1.85 (1.25-2.76)	.002	2.32 (1.58-3.41)	<.001	1.85 (1.04-3.30)	.04	1.27 (0.87-1.85)	.22	1.54 (1.04-2.27)	.03	1.28 (0.88-1.87)	.19
Nocturnal sleep duration at enrollment (h)	0.92 (0.79-1.06)	.23	0.88 (0.76-1.01)	.07	0.91 (0.79-1.04)	.17	0.94 (0.78-1.14)	.54	0.95 (0.83-1.09)	.47	0.92 (0.80-1.06)	.26	0.90 (0.79-1.04)	.15
Nighttime awakenings at enrollment (no. of awakenings)	1.14 (0.97-1.34)	.11	1.06 (0.91-1.25)	.44	1.18 (1.01-1.39)	.04	1.00 (0.81-1.25)	.98	1.09 (0.93-1.27)	.31	1.13 (0.97-1.32)	.13	1.10 (0.93-1.29)	.26
Parent-reported sleep problem at enrollment (none/small problem/very serious problem)	1.11 (0.73-1.67)	.63	1.27 (0.85-1.90)	.25	0.87 (0.58-1.30)	.49	1.21 (0.69-2.10)	.50	0.95 (0.64-1.42)	.80	0.84 (0.56-1.26)	.40	0.98 (0.66-1.46)	.92

*OR*, Odds ratio.

∗ Food-specific IgE (≥0.1 kU/L) to any food for EIG overall nonadherence and to the specific food for individual food-specific nonadherence. If sensitization status was included in the model based on skin prick test response at enrollment, the result was statistically nonsignificant for overall nonadherence and food-specific nonadherence to any individual food, with the exception of milk (positive milk skin prick test response: OR, 13.8; 95% CI, 1.68-112; *P* = .01). For each outcome, all the variables listed were included in the same logistic regression model.

Nonwhite ethnicity, increased maternal age, and lower enrollment maternal quality of life (psychological domain) all remained significantly related to increased EIG overall nonadherence. There was no statistically significant relationship with any measure of eczema at enrollment, be this visible eczema, eczema severity (SCORAD severity group), or SCORAD itself, although all odds ratios were greater than 1.0.

### Enrollment factors associated with EIG food-specific nonadherence (multivariable analysis)

Models were created for each individual food (Table II, other columns). Results were broadly similar to overall nonadherence. Ethnicity and maternal age were most strongly associated with nonadherence. Enrollment eczema SCORAD was significantly related to sesame and fish nonadherence but not to other foods. In contrast to univariate associations, once potential confounding factors were adjusted for, the relationship between enrollment sensitization to any individual food and subsequent nonadherence to these foods was attenuated (peanut odds ratio of 1.18 [95% CI, 0.39-3.59; *P* = .77] and egg odds ratio of 2.32 [95% CI, 0.85-6.31; *P* = .10]) and not statistically significant. Among the other variables, nighttime waking frequency was significantly associated with egg nonadherence.

### Postenrollment factors associated with nonadherence (univariate analysis)

**Fig 3:**
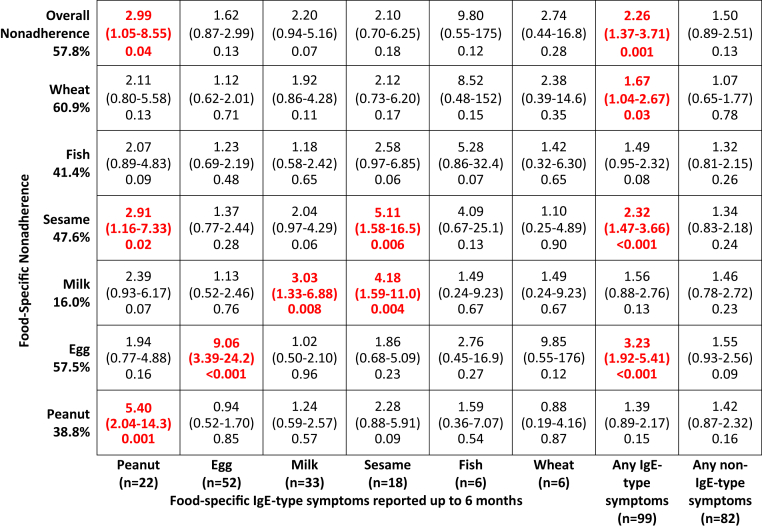
Reporting in the key early introduction period (up to 6 months) of IgE-type symptoms to specific foods, IgE-type or non–IgE-type symptoms to any of the early introduction foods, and the association with food-specific and overall per-protocol adherence. Penalized logistic regression of the association between symptoms with consumption of the 6 allergenic foods and food-specific and overall nonadherence. Symptoms manifesting by 6 months of age are presented for IgE-type symptoms for each specific food, IgE-type symptoms to 1 or more of the 6 early introduction foods, and non–IgE-type symptoms to 1 or more of the 6 early introduction foods.

We explored 3 postenrollment factors in the key early introduction period up to 6 months of age to assess their association with nonadherence in the EIG (see Table E6 in this article's Online Repository at www.jacionline.org). These were new onset of parent-reported eczema after enrollment but before 6 months, maternal reporting of feeding difficulties at the very beginning of solid food introduction (assessed at 4 months of age, see Table E7 in this article's Online Repository at www.jacionline.org), and reporting of IgE-type and non–IgE-type symptoms with consumption of early introduction foods before 6 months of age (see Fig E7 and Table E8 in this article's Online Repository at www.jacionline.org). Although new-onset eczema showed no association with overall or food-specific adherence, there was a strong association between adherence and early reported feeding difficulties and between adherence and the reporting of IgE-type symptoms to the early introduction foods in the key early introduction period (Fig 3). The 3 factors are reviewed in detail in the “Postenrollment factors associated with nonadherence” section in the Results section in this article's Online Repository.

The association between reporting of IgE-type and non–IgE-type symptoms and development of food allergy is reviewed in the “Parent-suspected IgE-type or non–IgE-type symptoms with food introduction in the EIG and the effect on food allergy” section in the Results section and Fig E8 in this article's Online Repository at www.jacionline.org.

### Targeted intervention to reduce the overall food allergy burden

**Fig 4:**
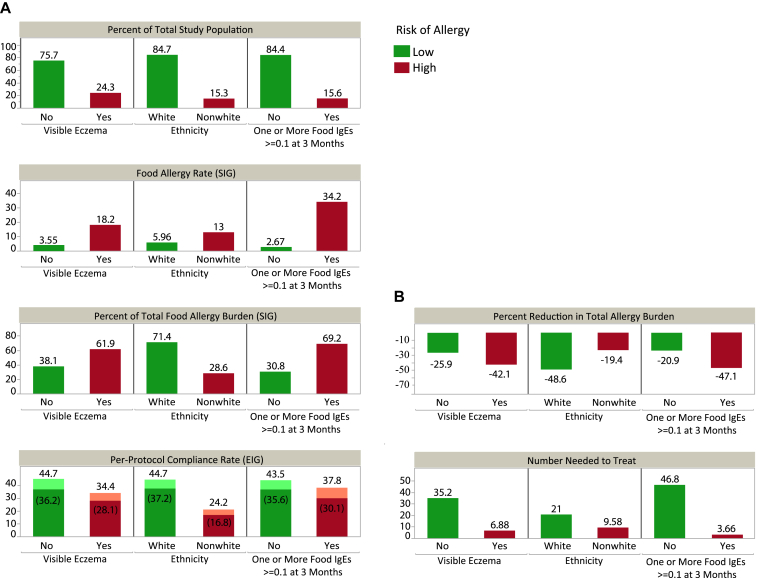
Contributions of subgroups to the proportion of food allergy cases in the SIG. **A,***Bar charts* provide prevalence calculations used to estimate the reduction in total allergy burden and number needed to treat. Per-protocol adherence rates are shown for those EIG participants whose adherence rates were evaluable and also as a proportion of the whole EIG (percentages in parentheses). **B,** An 80% treatment effect and 85% adherence across all risk factors for allergy is assumed. For example, infants in EAT with visible eczema comprised 61.9% of the total food allergy burden. Hypothetically, if per-protocol adherence could be achieved in 85% of this subgroup, then 52.6% (61.9%*85%) of the allergic burden would experience the intervention. Moreover, if an intervention effect of 80% is assumed, then the total reduction in food allergy that would be realized from intervening on this subgroup would be 42.1% (52.6%*80%).

The EAT study SIG participants can be used to explore the natural history of the development of food allergy independent of the early food introduction intervention. Children with visible eczema or food sensitization at enrollment or those of nonwhite ethnicity, although representing a small subgroup of the overall population, contributed disproportionately to the burden of overall food allergy (Fig 4, *A*, and see Fig E9 in this article's Online Repository at www.jacionline.org).

SIG participants with eczema at enrollment made up 24.3% of the study population but were responsible for 61.9% of the food allergy cases. Likewise, 15.6% of the SIG were sensitized on IgE testing at baseline to 1 or more foods but were responsible for 69.2% of food allergy cases in the SIG. Of the SIG, 15.3% were of nonwhite ethnicity but accounted for 28.6% of food allergy cases in the SIG, and a significantly greater proportion of nonwhite participants had visible eczema and/or IgE food-specific sensitization at enrollment (see Table E5 and Fig E9). Furthermore, in the EIG adherence was significantly lower in those participants of nonwhite ethnicity (Fig 4, *A*).

We modelled the effects of improved adherence in infants at high risk of developing food allergy (nonwhite infants, those with enrollment eczema, or those with enrollment sensitization [≥0.1 kU/L]) and how this might affect the prevalence of food allergy (Fig 4, *B*). Certain assumptions were made: an 80% intervention effect was assumed given the high efficacy observed in the EAT per-protocol analysis and the efficacy seen in the Learning Early About Peanut Allergy (LEAP) study intention-to-treat analysis.^14^ Assuming that a greater level of adherence could be achieved in these subgroups at high risk of developing food allergy than the 42% observed in the adherence-evaluable EIG children, we determined what the effect might be if an adherence rate of 85% is assumed. Fig 4, *B*, displays the percentage reduction in total allergy burden and the number needed to treat within the different subgroups.

Targeting the intervention to the group of nonwhite and/or eczematous infants would comprise a high-risk population of 71.4% of the food allergy burden. If 50%, 75%, or 85% adherence rates can be achieved in this high-risk group, reduction in the overall burden of food allergy in the whole population would be approximately 29%, 43%, and 49%, respectively.

## Discussion

Whilst 5.6% of EIG participants had a food allergy in the EAT study, 58% were nonadherent with the early introduction protocol. Hence the nonadherence rate was 10-fold greater than the food allergy rate. Notably, the nonadherence rate also significantly exceeded the prevalence rate of risk factors associated with developing a food allergy: visible eczema at enrollment (25%), enrollment sensitization to 1 or more foods on specific IgE testing (16%), and nonwhite ethnicity (15%).

In univariate analysis nonwhite ethnicity, older maternal age, lower enrollment maternal quality-of-life scores, and increasing SCORAD were associated with nonadherence to the EIG protocol. The enrollment factors that were found to remain significantly associated with nonadherence in the adjusted analysis were nonwhite ethnicity, older maternal age, and lower enrollment maternal psychological quality of life. Nonwhite ethnicity and older maternal age were associated in both study groups with postponing the introduction of allergenic foods, compromising the ability to be per-protocol adherent because the window for per-protocol–defined adherence was so short. This delay being present in the SIG could have been anticipated, having been reported in the Infant Feeding Survey undertaken in 2010 (Infant Feeding Survey 2010),^15^ and considered in more detail in the “Comparison with the Infant Feeding Survey 2010 findings” section in the Discussion section in this article's Online Repository at www.jacionline.org. However, unexpectedly, this delay was present in the EIG as well, despite all EIG families having being asked to introduce the allergenic foods as rapidly as possible.

After enrollment, although the reporting of IgE-type symptoms in EIG participants in the key early introduction period was common (16%), this too fell far short of the 58% nonadherence rate. Furthermore, the great majority of EIG participants reporting such symptoms were neither sensitized to any food at enrollment (76%) nor had a food allergy (82%). The strongest association with nonadherence was the early emergence of feeding difficulties, with 40% of non per-protocol EIG families reporting some or great difficulty feeding their infant at 4 months of age compared with 20% of the per-protocol EIG families.

Enrollment eczema was not associated with overall adherence in the adjusted analyses (although it was associated with food-specific adherence to sesame and fish). This is likely to reflect the mild phenotype of infants with eczema in the EAT study. Of the 160 EIG infants with enrollment eczema, in 123 it was mild (SCORAD <15), in 31 moderate (SCORAD 15 to <40), and in only 6 was it severe (SCORAD ≥40). Having eczema in childhood is strongly associated with dietary restriction and a reluctance to include allergenic foods in the diet. In a study of 100 children attending a pediatric dermatology clinic, 75% were having some form of parent-instigated dietary exclusion, and allergenic foods, including dairy products, eggs, and cow's milk, were being omitted by 48%, 27%, and 25%, respectively.^16^

It might have been anticipated that enrollment sensitization could lead to subclinical symptoms, resulting in a subclinical form of pre-existing food allergy that prevented adherence. This was not the case. Enrollment sensitization was not associated with overall or food-specific adherence. Furthermore, the EAT intervention was effective in an intention-to-treat analysis in the enrollment-sensitized EIG infants.^13^

The early emergence of parentally perceived feeding difficulties and aversive feeding behavior by 4 months of age, when families had only just started introducing solids to their EIG infants, had the strongest associations with nonadherence in the EIG and were also associated with later introduction of allergenic foods. This finding suggests that families were trying to feed their infants the allergenic food, but if the family perceived that the infant was not ready or mature enough and they were struggling to achieve the study's stipulated consumption levels, then, particularly for certain foods and having reached a specific level of consumption in the key early introduction period, they did not attempt to escalate this level further beyond 6 months.

That infant maturity might be a key factor was demonstrated in our recent analysis of sleep data within the cohort. In this study we showed in an intention-to-treat analysis that early introduction in the EIG was associated with greater duration of sleep and fewer night wakings, as well as significantly less parent-reported very serious sleep problems.^17^ Notably, EIG infants who were sleeping better at enrollment were more likely to subsequently be per-protocol adherent, suggesting that infant maturity is the link between the ability to sleep and eat better.^17^

Feeding difficulties were also shown in the Infant Feeding Survey 2010 to have a strong association with ethnicity (considered in more detail in the “Comparison with the Infant Feeding Survey 2010” section findings in the Discussion section in this article's Online Repository).^15^ However, survey questions about feeding difficulties in the context of a randomized controlled trial in which the intervention sets high expectations for the introduction of foods are likely to overestimate the prevalence of true feeding difficulties, which might be seen if a parent were left to introduce solids at a time and in a way of their choosing.

The primary strength of this analysis is that the EAT study cohort was recruited from the general population. The children were meticulously studied and have been shown to have demographic characteristics broadly similar to those of the population of England and Wales.^1^ The potential weakness of our findings is the extent to which it can be concluded that factors pertaining to the difficulty in following the highly prescriptive EAT early introduction regimen might relate to the success of early allergenic food introduction in the real world. However, we have previously shown with modelling of our consumption data that mean weekly consumption of 2 g of peanut protein (50% of the recommended EAT weekly dose) was associated with prevention of peanut allergy, and a dose-response relationship for protection against peanut allergy and egg allergy was apparent.^2^

It has been recognized by others that when clinical efficacy has been demonstrated in trials, such as EAT and particularly LEAP, translating this into a public health intervention is complex, and the results are likely to be subject to effect modification in different populations.^18^ Hence the call for plausibility trials to evaluate the effect of large-scale public health programs. Such trials serve to identify the barriers and facilitators of the intervention in the real world.^18^

Our findings suggest that there are certain groups who could benefit from directed support should the recommendation of early introduction of allergenic foods be adopted as a way to prevent food allergy. Specifically, the important factors were found to be nonwhite ethnicity, older mothers, and mothers with poorer quality of life. This also applies to infants with early-onset eczema in that although their adherence to per-protocol consumption of specific foods was not compromised compared with children without eczema, it is within this group that the majority of food allergy develops. Although these factors explain only a small part of the 58% of EIG families who were nonadherent, these groups of infants contributed disproportionately to the overall prevalence of food allergy in the EAT population. Indeed, our modelling shows that improved adherence in infants manifesting early eczema and from ethnic minorities raises the possibility of a substantial reduction in the burden of food allergy, with a 49% reduction if 85% adherence were achievable. Sufficiently high adherence rates with an early introduction regimen in these populations will be more challenging yet of great value.

The issue of ethnic differences in adherence to public health recommendations is well recognized.^19^ There is less of a literature on ethnic differences in adherence within the context of a randomized trial in which subjects have consented to enroll.

A recent study of 1000 expecting and 1000 new caregivers of infants less than 1 year of age reported questionable support for early allergenic solid food recommendations.^20^ However, it has been shown in Australia that updated guidelines issued in 2008 removing recommendations to delay allergenic solids have been associated with reduced delay in parents introducing egg and peanut into the diet.^21^ Cultural factors might well be important. Although US caregivers can perceive early peanut introduction to be difficult, the majority of Israeli infants are eating 2 g of peanut protein per week without the support of guidelines or a public health campaign.^22^

A number of countries, including the United States,^23^ Australia,^24^ and the United Kingdom,^5^, ^25^, ^26^ have issued new infant feeding guidelines in light of the EAT and LEAP study findings. Where a public health policy of early allergenic food introduction is being recommended, a significant amount of public health support is likely to be necessary to help specific groups at risk of low adherence in order to achieve a substantial reduction in the prevalence of food allergy.

**Clinical implications**

Nonwhite families, those with older mothers, and those with infants with early reported feeding difficulties or early-onset eczema would benefit from support to achieve early and sustained allergenic food consumption.

## Supplementary data

Online Repository text

Legends for Figs E1-E9

Fig E1

Fig E2

Fig E3

Fig E4

Fig E5

Fig E6

Fig E7

Fig E8

Fig E9
