# Supplementary material for: Factors influencing adherence in a trial of early introduction of allergenic food
Source: J Allergy Clin Immunol. 2019 Dec;144(6):1595–605. doi: 10.1016/j.jaci.2019.06.046 (PMC6904906; doi:10.1016/j.jaci.2019.06.046)
Supplement: Online Repository text [file mmc1.doc]

**Supplementary Appendix**

**Factors influencing adherence in a trial of early introduction of allergenic food**

**Michael R. Perkin, PhD,**^a^ **Henry T. Bahnson, MPH,^b^ Kirsty Logan, PhD,**^c^ **Tom Marrs, MB BS,^c^ Suzana Radulovic, MD,^c^ Rebecca Knibb, PhD,^d^ Joanna Craven, MPH,^c^ Carsten Flohr, PhD,^e^ E. N. Mills, PhD^f^, Serge A. Versteeg,^g^ Ronald van Ree, PhD,^gh^ and Gideon Lack, M.B. B.Ch,**^c^ **on behalf of the EAT Study Team**

From ^a^the Population Health Research Institute, St George's, University of London, ^b^the Benaroya Research Institute, Seattle, ^c^ the Paediatric Allergy Research Group Department of Women and Children’s Health, School of Life Course Sciences, King’s College London, ^d^the Department of Psychology, Aston University, ^e^the Unit for Population-Based Dermatology Research, St John’s Institute of Dermatology, School of Basic and Medical Biosciences, Faculty of Life Sciences & Medicine, King’s College London, ^f^the School of Biological Sciences, Division of Infection, Immunity and Respiratory Medicine, Manchester Academic Health Science Centre, Manchester Institute of Biotechnology, University of Manchester, ^g^the Department of Experimental Immunology, Academic Medical Center, Amsterdam; ^h^the Department of Otorhinolaryngology, Academic Medical Center, Amsterdam

**Conflict of interest:** none

**Methods**

**EIG early introduction regimen**

The EAT Study tested the hypothesis that the early introduction of multiple allergenic foods would generate an immunologically tolerizing milieu through the induction of regulatory mechanisms.

The mean age of enrollment onto the EAT study was 14.7 weeks.^1^ Families in the EIG had to await the return of safety blood results before consumption commenced (up to four days after enrollment). EIG families were then told to first introduce nonallergenic foods such as baby rice and/or pureed fruits or vegetables during the first week until they were established on solid food. They then continued with these solids and additionally introduced cow’s milk yogurt on two days of the second week. Hence, median age of introduction of dairy in the EIG was 17.3 weeks.^1^

During weeks three and four of the early introduction regimen, peanut, hard-boiled egg, sesame and whitefish (cod) were introduced sequentially in a random order with two new foods introduced per week. Finally, wheat was introduced in week 5, reflecting the guidance on optimal timing of wheat introduction after 4 months of age, and by week 6, infants were ideally consuming the required amount of all 6 allergenic foods each week.

Randomisation was effective and the median age of introduction of the four randomised foods was 19.6 weeks. Wheat, which was always introduced last and not before four months of age had a median age of introduction of 20.6 weeks.^1^

Food vehicles chosen for introducing the allergen to the infants were those which families would be familiar and comfortable with using. Yoghurt was used for introducing cow's milk protein. We specifically excluded recommending using cow's milk formula, which might have affected breastfeeding. Boiled egg was used instead of egg power. Wheat was introduced as a wheat based breakfast cereal (Weetabix), widely consumed in the UK and suitable for infants as it dissolves in milk to form an easily consumed paste. Peanut butter has historically not commonly been given to UK infants, but is the vehicle that virtually all parents of infants would chose, if they were feeding their infant peanut. The only food that is rarely given to infants, and which many parents were not familiar with, was tahini (ground sesame paste), although hummus (the ingredients of which include tahini) is consumed by many children.

Participants were asked to consume the equivalent of 2 g of each allergenic food protein twice each week (4 g of allergen protein per food per week). Therefore the full weekly amount for the allergenic foods consisted of 2 small 40- to 60-g portions of cow’s milk yogurt, 3 rounded teaspoons of peanut butter, 1 small hard-boiled egg (<53 g), 3 rounded teaspoons of sesame paste, 25 g of whitefish, and 2 wheat-based cereal biscuits (eg, Weetabix).

**Information collected**

*Online Questionnaire data*

Infant sleep was assessed in every online questionnaire through to two years and again at three years of age using the Brief Infant Sleep Questionnaire (BISQ).^2^ Maternal quality of life was recorded using the WHO Quality of Life BREF questionnaire at three time points: enrollment, one year and three years of age. This is a shorter version of the WHOQOL-100 and includes 26 questions. Two enquire about overall quality of life and general health. The remaining 24 questions are divided into four domains of quality of life: Physical health (7 items: Activities of daily living; Dependence on medicinal substances and medical aids; Energy and fatigue; Mobility; Pain and discomfort; Sleep and rest; Work Capacity), Psychological (6 items: Bodily image and appearance; Negative feelings; Positive feelings; Self-esteem; Spirituality/Religion/Personal beliefs; Thinking, learning, memory and concentration), Social relationships (3 items: Personal relationships; Social support; Sexual activity), and Environment (8 items: Financial resources; Freedom, physical safety and security; Health and social care: accessibility and quality; Home environment; Opportunities for acquiring new information and skills; Participation in and opportunities for recreation/leisure activities; Physical environment - pollution/noise/traffic/climate; Transport).^3^ Four domain scores can be generated denoting an individual's perception of quality of life in each particular domain. Domain scores are scaled in a positive direction (i.e. higher scores denote higher quality of life). Scores range from 4 to 20. There were no significant differences in quality of life scores in each of the four domains between the two groups at enrollment. Poorer psychological domain quality of life was defined as less than the median score (16).

The online questionnaires allowed families to report any suspected symptoms with food ingestion, and to list suspected foods through responding to a generic question: “Does your child have adverse reactions to any foods, such as eczema, breathing problems or gastrointestinal problems?” If they answered yes, they were asked to describe the problems and identify the suspected food/foods. The answers to these questions were coded by food and symptom type, with clinical judgement used to distinguish between IgE-type symptoms (urticaria, angioedema, acute rash, respiratory symptoms, acute vomiting), and non-IgE-type symptoms (diarrhoea, constipation, reflux, delayed vomiting, delayed rash, eczema). These data were then used to give an indication of the prevalence of parent reported IgE and non-IgE-type food symptoms to specific foods and to grouped foods. Foods were grouped as any of the six early introduction foods, any non-early introduction food, or any food.^4^

Feeding difficulty and aversive feeding behaviour were enquired about at 4, 6, 9 and 12 months and two and three years of age. Questions used were based on previously published work on taste and sensory aversions in adults and were adapted for use in babies and young children.^5^ In this paper, the effect on adherence of symptoms reported at the earliest time point, four months, is assessed and the key question ascertained was whether the family had experienced: "Any difficulty feeding baby what you want them to eat?"

*Clinic visit data*

Scheduled clinic visits took place at enrollment, one and three years of age. All children were examined for eczema at all three clinic visits, using the UK diagnostic criteria-based photographic protocol of the International Study of Asthma and Allergies in Childhood (ISAAC) Phase Two.^6^ Disease severity was determined by the Scoring Atopic Dermatitis (SCORAD) index.^7^ SCORAD was categorized as mild (<15), moderate (15 to less than 40), and severe (40 or more).^7^ Transepidermal water loss (TEWL) was measured using the Biox Aquaflux® AF200 closed condenser chamber device on the unaffected skin of the volar aspect of the forearm 2009.^8^

Families of participants could report perceived problems with food consumption via a study dedicated telephone line, a study email address or in an interim questionnaire. Any family reporting food aversion or refusal, or a suspected food allergy, were invited to attend an unscheduled clinic visit where the participant was assessed with skin-prick testing, followed by a food challenge where necessary.

**Restricted window to achieve per-protocol status**

Per-protocol criterion C for the EIG proved to be particularly challenging (Table E1). The window in which to achieve 5 weeks of 75% consumption of a specific allergenic food by 26 weeks of age (6 months), particularly for the four randomized foods, was narrow, given the average age of first consumption of these foods was 19.6 weeks. For wheat, the window was shorter still, as wheat consumption always commenced last (median age of introduction of 20.6 weeks) and consumption was not allowed before four months of age.

**Results**

**EIG adherence nonevaluable participants**

Overall adherence status could only be determined in 81% (529/652) of the EIG participants (Table E1). In the remaining 19% (123/652) of the EIG participants, their adherence could not be determined because insufficient questionnaires had been completed in the key early introduction period (the 4, 5 and 6 month questionnaires). We have previously demonstrated that levels of consumption of the allergenic foods in the overall adherence nonevaluable EIG participants (red line) were similar to the overall nonadherent EIG participants (green line) (Fig E1, left panels).^1^

However, food-specific adherence levels varied considerably, ranging from 39% for wheat, to 84% for milk (Table E3). When we assessed weekly food consumption levels by food-specific adherence status (Fig E1, central panels), a different pattern was observed. Compared with the left hand panels, the most notable observation is that the weekly consumption levels in the food-specific nonadherent groups (red line) are at significantly lower levels than those observed in the overall nonadherent group (red line) in the left hand panels. Furthermore, predominantly in the key early introduction period (up to six months of age), weekly consumption levels for certain foods in the nonevaluable group (green line) were much closer to the levels observed in the per-protocol group (blue line) (particularly for peanut and sesame).

This was even more apparent when we analyzed the proportion of EIG participants who were consuming 75% or more of the recommended dose of each early introduction food. (Fig E1, right panels). Participants whose adherence status was nonevaluable for specific foods (green line) were consuming the specific food at or above the per-protocol threshold much more frequently than the food-specific non per-protocol participants (red line).

In Fig E2 we explore the issue of how many foods were being consumed at or above the per-protocol threshold by overall compliance status. The number of foods being consumed at or above the per-protocol threshold at any particular time point is similar between the overall noncomplied group (red line) and the overall compliance nonevaluable group (green line), other than the data from the 6 month interim questionnaire showing a higher level of consumption in the overall non evaluable group (green line) (Fig E2, Panel A). However, Panel A represents the mean number of foods being consumed at a particular time point, and therefore does not provide any information about how many participants might have been exceeding the overall per-protocol definition of five or more foods being consumed at 75% or more of the recommended dose. This is presented in Panel B and in the key early introduction period, a higher percentage of EIG participants in the overall adherence nonevaluable group (green line) would appear to be achieving this threshold, than in the overall noncomplied group (red line), particularly just before six months.

To have an adherence status that was nonevaluable was entirely related to insufficient interim questionnaires being completed to allow consumption levels to be determined in the key early introduction period (up to six months of age). Questionnaire completion rates in participants whose overall adherence status was nonevaluable continued to be significantly lower throughout the first year of life, compared with the other two groups. It is only those adherence nonevaluable families who completed an interim questionnaire at any specific monthly time point up to one year of age that provide the consumption data for any specific month to compare with the other two groups that is presented in Figs E1 & E2. Therefore, in Fig 2, Panel C, it can be seen that whilst the overall adherence nonevaluable group includes 123 EIG participants, the monthly interim questionnaire completion rates through to one year in this group were very low. The data points for most months between 4 and 12 months are based on less than 30 respondents. Thus considerable caution has to be exercised as to how representative the 30 or so families that were completing these interim questionnaires are of the whole group of 123 overall nonevaluable EIG participants.

When comparing the demographic characteristics of the EIG overall nonadherent and adherence nonevaluable groups, the two groups were broadly similar, for example with both having much higher proportions of nonwhite participants (Table E2). However, there were some important differences, eczema was significantly less common in the adherence nonevaluable EIG group (Table E2).

Whilst these results suggest that the adherence nonevaluable group's consumption levels were more similar to the adherent group, particularly in the key early introduction period, because the adherence nonevaluable group's precise adherence status was unknown, we undertook the analyses in this paper comparing the per-protocol and non per-protocol groups. We did, however, repeat the analyses with the non per-protocol and the adherence nonevaluable groups combined. The effect of eczema on nonadherence diminished and the effect of ethnicity increased but the results were in general broadly similar.

**Association between ethnicity and baseline demographic characteristics**

Given that ethnicity was such an important determinant of nonadherence, we explored whether differences existed in other enrollment demographic characteristics between ethnic groups (Table E5). Asian, black and Chinese participants were more likely to come from families with siblings and more likely to have a sibling with a parent-reported history of food allergy. Smoking rates, both in mothers and fathers, were highest in the mixed ethnicity participants. Birth weight significantly diminished across the ethnic categories, with Asian, black or Chinese participants having the lowest birth weight. With regards to atopy there was a stepwise increase in visible eczema across ethnic groups with it being highest in the Asian, black or Chinese group. The eczema that was present was also more severe for the Asian, black or Chinese participants compared with the other two groups. The trend in visible eczema was matched by a similar increase in sensitisation rates at enrollment in the EIG with it being 3.6% in the white participants but 23.4% in the Asian, black or Chinese participants. Conversely, the opposite trend was seen with regards to parental atopy. Maternal asthma, maternal and paternal atopy all showed a stepwise reduction from white, to mixed, to Asian, black and Chinese participants. Maternal eczema was also significantly lower in the latter group compared with the other two groups. Maternal quality of life scores for three of the four domains (physical, social and environment) were significantly lower in the mixed group and for two domains (physical and environment) in the Asian, black or Chinese mothers, compared with white mothers.

**Enrollment factors associated with overall nonadherence (univariate analysis)**

Per-protocol adherence in the EIG could be compromised by three independent factors: delayed introduction of allergenic foods resulting in insufficient time to achieve five or more weeks consumption at the requisite amount between enrollment and six months of age, insufficient amount of an allergenic food being consumed (<75% of the recommended amount), and an insufficient total number of foods being consumed at the recommended amount (less than five of the six early introduction foods).

*Nonadherence - delayed introduction of allergenic foods*

Nonwhite ethnicity and increased maternal age both showed independent and statistically significant associations with postponed introduction of solids into the infant's diet. For the former, the difference between white participants and the Asian, black or Chinese infants amounted to 5.9 days in the SIG (p=0.15) and 6.4 days (p=0.005) in the EIG. For the latter, the difference in age of solid food introduction when comparing a 20 year old mother with a 45 year old mother amounted to 12.5 days in the SIG and 7.5 days in the EIG.

*Nonadherence - insufficient amount of the allergenic foods consumed*

Nonwhite ethnicity, increased maternal age and enrollment eczema severity (SCORAD category) were all associated with EIG infants being fed less than the recommended weekly dose of allergenic foods. This reduced consumption persisted throughout the first year of life for several of the foods.

*Nonadherence - insufficient total number of foods*

The reduction in consumption of individual allergenic foods meant that for nonwhite ethnicity, maternal age and eczema severity (SCORAD category) there was a concomitant reduction in the total number of foods being consumed at the per-protocol level (Fig E4, left hand panels). Particularly for nonwhite participants this reduction persisted throughout the first year of life.

This, in turn, affected the ability of certain subgroups to fulfil the per-protocol definition of adherence on any particularly week, i.e. on a specific week to have consumed five or more of the six foods, at 75% or more of the recommended dose (Fig E4, right hand panels). Again, for ethnicity the effect was particularly marked with 80% of white participants achieving the overall per-protocol threshold from shortly after six months of age, whilst 60% of mixed ethnicity participants were meeting the threshold beyond six months, the figure dropping to around 50% of black, Asian or Chinese participants.

**Enrollment factors associated with food-specific nonadherence (univariate analysis)**

Factors influencing overall nonadherence in the EIG were similar for individual foods (Table E4). Nonadherence was statistically significantly related to the presence of any visible eczema at enrollment for several of the individual foods.

A strong association with maternal older age was observed for food-specific nonadherence for four of the six foods and for paternal older age with egg and fish non per-protocol adherence. Baseline maternal quality of life factors also appeared significant, particularly for peanut with mothers with lower quality of life scores being significantly more likely to be non per-protocol.

Significant associations with non per-protocol status were also seen with several measures assessed in the BISQ sleep questionnaire completed at enrollment. Increased frequency of night wakings was significantly associated with non per-protocol adherence specifically for peanut and egg. Infants who were sleeping in the parent’s bed at enrollment were significantly more likely to be non per-protocol adherent whilst those who were put to bed alone were significantly less likely to be nonadherent.

**IgE-type symptom reporting and enrollment sensitization**

There was a statistically significant relationship between being sensitized to a specific food at enrollment and subsequently reporting symptoms to the food. For example, 18% (4/22) of EIG participants sensitized to peanut at enrollment reported IgE-type symptoms with peanut introduction, compared with 3.2% (17/533) of non-peanut sensitized EIG families reporting symptoms with peanut introduction (p<0.001). However, it important to note that 18 out of the 22 peanut sensitized children therefore did not report any symptoms with peanut introduction.

IgE-type symptoms were reported with peanut consumption by coincidentally the same number of EIG children (22). One of these did not have IgE data. It is equally important to note that of the remaining 21 children, 17 were not sensitized to peanut at enrollment.

Both the number of EIG participants with reported peanut symptoms (22) and the number sensitized to peanut at enrollment (22) significantly exceeded the number of EIG participants who ultimately developed a peanut allergy (7).

**Consumption of each allergenic food by enrollment specific IgE sensitization status**

Enrollment specific IgE sensitization (0.1kU/l or greater) did not have a significant effect on the ability to consume milk, sesame or wheat at the 75% recommended dose threshold (Fig E6). No infant was sensitized (0.1 kU/l or greater) to fish at enrolment. For peanut and egg there was a delay in introduction in the key early introduction period in specific IgE sensitized infants compared with the non-sensitized infants. This resulted in a significant univariate association between enrollment IgE sensitization and food-specific nonadherence for egg and the relationship was of borderline significance for peanut (Fig 2). This was likely to be a consequence of the study design. A significant proportion of specific IgE sensitized EIG infants were also skin prick test positive to the same food at enrollment, particularly for egg (Fig E6 - numbers in brackets). Any EIG infant who was enrollment skin prick test positive to an early introduction food came back for a supervised food challenge before being allowed to start consuming this food. They were issued with an amended early introduction regimen with the sensitized food being introduced last (so the scheduling of the challenge did not defer commencing consumption of the other early introduction foods). Inevitably this meant there was a delay before they could commence introducing the food into their diet, reflected in the figure.

Interestingly the difference was not apparent for milk despite there being 10 EIG milk IgE sensitized infants who were also skin prick test positive to milk. These specific families would appear to have been able to rapidly increment their infant's milk consumption once they were allowed to commence consumption such that in the week before turning six months of age these 10 infants were consuming 81% of the recommended weekly milk dose. In contrast, at the same point in time, the 6 peanut skin prick and specific IgE positive infants were consuming 50% of the peanut weekly dose and the 22 egg skin prick and specific IgE positive infants were consuming 62% of the egg weekly dose.

**Post enrollment factors associated with nonadherence**

*Post enrollment - Parent early reported feeding difficulties*

The strongest relationships with non per-protocol adherence in the EIG were observed between the questions that assessed the early emergence of parent reported feeding difficulties and food refusal at 4 months of age (Table E6). The reporting by parents of feeding difficulties at 4 months of age was associated with sustained lower levels of consumption of the six allergenic foods, significantly compromising the number of foods being consumed at the per-protocol level (Fig E4, left hand panel) and the ability to meet the overall per-protocol threshold (Fig E4, right hand panel).

Whilst the associations between food aversion behaviours and non per-protocol status were notable for the consistency of highly statistically significant relationships, there was no evidence that these behaviours were significantly associated with the development of food allergy in the EIG (Table E7).

*Post enrollment - Parent suspected IgE or non-IgE-type symptoms with early food introduction in the EIG and the effect on food-specific and overall adherence*

Rates of reporting food-specific symptoms during the early introduction period varied significantly by food (Table E8). Given that there were 652 infants in the EIG, absolute numbers of families reporting any symptoms ranged from very low, with 7 reports of any symptoms for fish (1.1% of the EIG cohort), to 68 for egg (10.4%). For egg, IgE-type symptoms predominated (52 versus 28 reporting non-IgE-type symptoms). Milk had a similar number of families reporting any symptoms (63), but the situation reversed with non-IgE-type symptoms predominating (46 versus 33). Compared to egg, peanut was perhaps surprisingly well tolerated, with only 27 reporting symptoms (22 IgE, 10 non-IgE). Any symptoms to one or more of the early introduction foods was reported by 149 families (22.9% of the whole EIG).

IgE-type food-specific symptoms were associated with nonadherence to the same food, with the exception of peanut which also influenced nonadherence to sesame, and for sesame which also influenced nonadherence to milk (Fig 3 & Table E6). For reported IgE-type symptoms with both peanut and sesame, the odds ratios for nonadherence to other foods were higher than those seen for IgE-type symptoms with egg and milk, suggesting that symptoms to peanut and sesame were more likely to make a family concerned about introducing the other early introduction foods.

IgE-type symptoms to one or more of the six early introduction foods was strongly associated with food-specific and overall nonadherence, whereas no statistically significant associations with adherence were seen for non-IgE-type symptoms to one or more of the early introduction foods. Milk was the only food for which the reporting of non-IgE-type symptoms was associated with milk non per-protocol adherence (Table E6).

EIG families who were overall nonadherent were significantly more likely to have reported IgE-type symptoms in the key early introduction months than either EIG per-protocol infants or SIG infants (Fig E7). In fact, per-protocol EIG families and SIG families reported similar rates of IgE-type symptoms when allergenic foods were first introduced into the diet, but the former then showed particularly low rates of symptom reporting throughout the rest of the first year of life.

**Parent suspected IgE or non-IgE-type symptoms with food introduction in the EIG and the effect on food allergy**

Reporting of food-specific IgE-type symptoms was associated with food allergy to that food (Fig E8). Relationships were food-specific. Both IgE and non-IgE-type symptoms to any of the six foods were strongly associated with the primary outcome. When the two factors were included in a model with an interaction term, both remained statistically significant, suggesting that the association of both variables with the primary outcome was not explained by the strong correlation between the two.

It is important to note that whilst there were strong food-specific correlations between the early reporting of symptoms and subsequent food allergy, the great majority of EIG infants in whom such symptoms were reported did not develop an allergy to that food. For example, 33 EIG families reported IgE-type symptoms to milk but only 2 EIG infants developed a milk allergy. One other EIG infant developed a milk allergy and they reported no IgE symptoms with milk in the early introduction period.

**Discussion**

**Comparison with the Infant Feeding Survey 2010 findings**

In the Infant Feeding Survey 2010 (IFS2010) there was also a very strong positive association between increasing maternal age and later introduction of solids (19% of mothers aged 35 or over had begun introducing solids by four months of age compared with 57% of mothers under 20). Similarly, with ethnicity, nonwhite mothers introduced solids significantly later. Nearly eight in ten (77%) white mothers had introduced solids by the time their baby was five months old compared with around two-thirds of mothers from Asian, Black, Chinese backgrounds.

In the IFS2010 Asian, Black, and Chinese mothers were most likely to report having experienced difficulties introducing solid foods to their baby (16%, 14% and 18% respectively versus 10% in white mothers). Asian and Black mothers were also most likely to report their child as being a fussy or faddy eater (both 9% versus 4% in white mothers).^9^

If feeding difficulties explained the greatest proportion of EIG nonadherence, the question arises as to what was responsible for the feeding difficulties. Associations with sleep parameters suggest that other aspects of the infant such as their maturity might well be significant. However historical data would suggest that young infants are clearly able to introduce solids very early: the proportion of UK infants given solids by eight weeks of age being 49% in 1975.^10^

**References**

1. Perkin MR, Logan K, Marrs T, Radulovic S, Craven J, Flohr C et al. Enquiring About Tolerance (EAT) study: Feasibility of an early allergenic food introduction regimen. J Allergy Clin Immunol 2016; 137(5):1477-86.

2. Sadeh A. A brief screening questionnaire for infant sleep problems: validation and findings for an Internet sample. Pediatrics 2004; 113(6):e570-e577.

3. Skevington SM, Lotfy M, O'Connell KA. The World Health Organization's WHOQOL-BREF quality of life assessment: psychometric properties and results of the international field trial. A report from the WHOQOL group. Qual Life Res 2004; 13(2):299-310.

4. Perkin MR, Logan K, Tseng A, Raji B, Ayis S, Peacock J et al. Randomized Trial of Introduction of Allergenic Foods in Breast-Fed Infants. N Engl J Med 2016; 374(18):1733-43.

5. Knibb RC, Smith DM, Booth DA, Armstrong AM, Platts RG, Macdonald A et al. No unique role for nausea attributed to eating a food in the recalled acquisition of sensory aversion for that food. Appetite 2001; 36(3):225-34.

6. Weiland SK, Bjorksten B, Brunekreef B, Cookson WO, von ME, Strachan DP. Phase II of the International Study of Asthma and Allergies in Childhood (ISAAC II): rationale and methods. Eur Respir J 2004; 24(3):406-12.

7. Kunz B, Oranje AP, Labrèze L, Stalder JF, Ring J, Taïeb A. Clinical Validation and Guidelines for the SCORAD Index: Consensus Report of the European Task Force on Atopic Dermatitis. Dermatology 1997; 195(1):10-9.

8. Farahmand S, Tien L, Hui X, Maibach HI. Measuring transepidermal water loss: a comparative in vivo study of condenser-chamber, unventilated-chamber and open-chamber systems. Skin Res Technol 2009; 15(4):392-8.

9. McAndrew F, Thompson J, Fellows L, Large A, Speed M, Renfrew MJ. Infant Feeding Survey 2010. 2012. Health and Social Care Information Centre.

10. COMA working group on the weaning diet. Weaning and the weaning diet. 1994. London.

**TABLE E1. Overall per-protocol adherence** criteria in the EAT study

| **Adherence definitions** | **Adherence evaluable children meeting the adherence definitions** |
| --- | --- |
| **Standard Introduction Group (SIG)**  *(N=606/651 children adherence evaluable)** |  |
| - Criterion A: Exclusive breastfeeding for at least three months duration (water and/or oral rehydration solution allowed) | 100% (606/606) (A)  12.0% have had water by 3 months of age |
| - Criterion B: Continued breastfeeding up to five months of age | 99.7% (604/606) (B) |
| - Criterion C: No consumption of peanut, egg, sesame, fish or wheat before five months | 97.4% (590/606) (C) |
| - Criterion D: No introduction of cow’s milk formula (or goat’s milk formula) (or consumption of less than 300 mls/day) between three months and six months of age | (1) No formula pre six months 85.6% (519/606)  (2) Consumption of less than 300mls/day 8.8% (53/606)†  (1) or (2) 94.4% (572/606) (D)  †median age of introduction of 22 weeks |
| **Overall SIG per-protocol adherence (meets all criteria)** | **92.1% (558/606) (A, B, C & D)** |
| **Early Introduction Group (EIG)**  *(N=529/652 children adherence evaluable)** |  |
| - Criterion A: Exclusive breastfeeding for three months duration (water and/or oral rehydration solution allowed) | 100% (529/529) (A)  13.1% have had water by 3 months of age |
| - Criterion B: Continued breastfeeding up to five months of age | 99.6% (527/529) (B) |
| - Criterion C: Consumption of at least five of the allergenic foods in at least 75% of the recommended amount (3g allergen protein/week), for at least five weeks between three months and six months of age | 42.3% (224/529) (C) |
| **Overall EIG per-protocol adherence (meets all criteria)** | **42.2% (223/529) (A, B & C)** |

* Adherence status nonevaluable for 7% (45/651) of the SIG and 19% (123/652) of the EIG participants

**TABLE E2. Univariate analysis of factors influencing EIG per-protocol status**

|  |  | **EIG Per-Protocol status** | | | | | | |
| --- | --- | --- | --- | --- | --- | --- | --- | --- |
|  |  | **Per-Protocol**  **(A)**  (N=223) | **Non-Per-Protocol**  **(B)**  (N=306) | **P value**  **(AvB)** | **Adherence Nonevaluable**  **(C)**  (N=123) | **P value**  **(AvC)** | **Non-Per-Protocol or Adherence Nonevaluable**  **(B+C)**  (N=429) | **P value**  **(Av(B+C))** |
| **Demography** |  |  |  |  |  |  |  |  |
| Sex (female) (%) |  | 49.3 | 53.6 | 0.33 | 52.0 | 0.63 | 53.2 | 0.35 |
| Ethnicity (%) | *White* | 92.8 | 83.7 | 0.002 | 76.4‡ | <0.001 | 81.6 | <0.001 |
|  | *Mixed* | 5.4 | 8.2 |  | 8.9 |  | 8.4 |  |
|  | *Black/Asian/Chinese* | 1.8 | 8.2 |  | 14.6 |  | 10.0 |  |
| Siblings (any) (%) |  | 59.6 | 64.1 | 0.30 | 65.0 | 0.32 | 64.3 | 0.24 |
| Pet ownership (any) (%) |  | 45.7 | 39.5 | 0.15 | 33.6 | 0.03 | 37.9 | 0.05 |
| Maternal education (%) | *>18 years* | 83.4 | 82.7 | 0.80 | 78.1 | 0.008 | 81.4 | 0.22 |
|  | *17-18 years* | 13.5 | 13.1 |  | 10.6 |  | 12.4 |  |
|  | *16 years* | 3.1 | 4.3 |  | 11.4 |  | 6.3 |  |
| Paternal education (%) | *>18 years* | 69.5 | 71.2 | 0.86 | 65.6 | 0.16 | 69.6 | 0.62 |
|  | *17-18 years* | 17.5 | 15.7 |  | 13.9 |  | 15.2 |  |
|  | *16 years* | 13.0 | 13.1 |  | 20.5 |  | 15.2 |  |
| **Smoking** |  |  |  |  |  |  |  |  |
| Maternal smoking (%) |  | 3.6 | 2.6 | 0.52 | 4.9 | 0.55 | 3.3 | 0.83 |
| Paternal smoking (%) |  | 11.2 | 10.5 | 0.78 | 10.7 | 0.88 | 10.5 | 0.79 |
| **Birth history** |  |  |  |  |  |  |  |  |
| Caesarean delivery (%) |  | 24.7 | 29.1 | 0.26 | 29.3 | 0.35 | 29.1 | 0.23 |
| **Infant weight** |  |  |  |  |  |  |  |  |
| Birth to enrollment weight gain | *Lowest quartile* | 29.2 | 24.5 | 0.55 | 22.1 | 0.56 | 23.8 | 0.44 |
|  | *2^nd^ quartile* | 25.1 | 23.9 |  | 26.2 |  | 24.5 |  |
|  | *3^rd^ quartile* | 22.9 | 24.8 |  | 25.4 |  | 25.0 |  |
|  | *Highest quartile* | 22.9 | 26.8 |  | 26.2 |  | 26.6 |  |
| Enrollment weight | *Lowest quartile* | 26.5 | 25.5 | 0.38 | 22.0 | 0.44 | 24.5 | 0.31 |
|  | *2^nd^ quartile* | 26.5 | 23.5 |  | 23.6 |  | 23.5 |  |
|  | *3^rd^ quartile* | 27.4 | 24.8 |  | 27.6 |  | 25.6 |  |
|  | *Highest quartile* | 19.7 | 26.1 |  | 26.8 |  | 26.3 |  |
| **Enrollment skin barrier** |  |  |  |  |  |  |  |  |
| Visible eczema at 3m visit (%) | *None* | 79.8 | 71.9 | 0.07 | 76.4 | 0.68 | 73.2 | 0.14 |
|  | *SCORAD 1-15* | 16.6 | 20.9 |  | 20.3 |  | 20.8 |  |
|  | *SCORAD >15* | 3.6 | 7.2 |  | 3.3 |  | 6.1 |  |
| Raised TEWL |  | 30.2 | 34.6 | 0.28 | 27.6 | 0.62 | 32.6 | 0.52 |
| **Enrollment sensitization** |  |  |  |  |  |  |  |  |
| Skin-prick positive at 3m visit (%) |  | 4.0 | 5.2 | 0.52 | 6.5 | 0.31 | 5.6 | 0.39 |
| Food-specific IgE (≥0.1 kU/l) |  | 13.6 | 16.6 | 0.36 | 17.3 | 0.38 | 16.8 | 0.31 |
| Food-specific IgE (≥0.35 kU/l) |  | 2.9 | 6.5 | 0.07 | 9.1 | 0.02 | 7.2 | 0.03 |
| **Eczema natural history** |  |  |  |  |  |  |  |  |
| New onset eczema (4-6m) (%) |  | 10.3 | 12.8 | 0.39 | 2.3 | 0.02 | 10.5 | 0.96 |
| Enrollment eczema or new onset eczema (4-6m) (%) |  | 30.5 | 40.9 | 0.02 | 27.9 | 0.66 | 38.0 | 0.06 |
| **Family atopy status** |  |  |  |  |  |  |  |  |
| Maternal atopy (%) |  | 60.1 | 64.7 | 0.28 | 58.2 | 0.73 | 62.9 | 0.49 |
| Paternal atopy (%) |  | 51.1 | 51.0 | 0.98 | 48.4 | 0.62 | 50.2 | 0.83 |
| **Maternal factors** |  |  |  |  |  |  |  |  |
| Physical QOL | *Highest quartile* | 26.9 | 17.1 | 0.04 | 20.4 | 0.24 | 18.0 | 0.04 |
|  | *2^nd^ quartile* | 19.6 | 26.9 |  | 26.2 |  | 26.7 |  |
|  | *3^rd^ quartile* | 27.9 | 29.0 |  | 22.3 |  | 27.3 |  |
|  | *Lowest quartile* | 25.6 | 26.9 |  | 31.1 |  | 28.0 |  |
| Psychological QOL | *Highest quartile* | 30.6 | 21.3 | 0.001 | 18.5 | 0.11 | 20.6 | 0.002 |
|  | *2^nd^ quartile* | 16.0 | 8.4 |  | 16.5 |  | 10.5 |  |
|  | *3^rd^ quartile* | 27.4 | 39.2 |  | 30.1 |  | 36.8 |  |
|  | *Lowest quartile* | 26.0 | 31.1 |  | 35.0 |  | 32.1 |  |
| Social QOL | *Highest quartile* | 25.1 | 20.0 | 0.31 | 14.6 | 0.10 | 18.5 | 0.17 |
|  | *2^nd^ quartile* | 14.6 | 16.1 |  | 11.7 |  | 14.9 |  |
|  | *3^rd^ quartile* | 38.8 | 36.4 |  | 45.6 |  | 38.9 |  |
|  | *Lowest quartile* | 21.5 | 27.5 |  | 28.2 |  | 27.7 |  |
| Environment QOL | *Highest quartile* | 30.1 | 20.0 | 0.07 | 23.3 | 0.09 | 20.9 | 0.07 |
|  | *2^nd^ quartile* | 21.0 | 22.5 |  | 24.3 |  | 22.9 |  |
|  | *3^rd^ quartile* | 27.9 | 33.3 |  | 20.4 |  | 29.9 |  |
|  | *Lowest quartile* | 21.0 | 24.2 |  | 32.0 |  | 26.3 |  |
| Maternal age (33+) (%) |  | 52.0 | 63.1 | 0.01 | 56.9 | 0.38 | 61.3 | 0.02 |
| **Paternal factors** |  |  |  |  |  |  |  |  |
| Paternal age (35+) (%) |  | 52.0 | 60.1 | 0.06 | 49.6 | 0.67 | 57.1 | 0.22 |
| **Childcare** |  |  |  |  |  |  |  |  |
| Nursery/childcare |  | 2.2 | 2.6 | 0.79 | 0.8 | 0.33 | 2.1 | 0.91 |
| **Infant sleep at enrollment** |  |  |  |  |  |  |  |  |
| Where does baby sleep | *Cot in separate room* | 23.3 | 17.8 | 0.06 | 13.6 | 0.01 | 16.7 | 0.01 |
|  | *Cot in parents’ room* | 66.2 | 67.8 |  | 66.0 |  | 67.4 |  |
|  | *In parents’ bed* | 8.2 | 13.6 |  | 19.4 |  | 15.2 |  |
|  | *Cot in sibling’s room* | 2.3 | 0.7 |  | 1.0 |  | 0.8 |  |
| How baby falls asleep | *While feeding* | 35.2 | 43.9 | 0.26 | 47.6 | 0.25 | 44.9 | 0.14 |
|  | *Being rocked* | 3.7 | 4.2 |  | 4.9 |  | 4.4 |  |
|  | *Being held* | 10.5 | 10.2 |  | 7.8 |  | 9.5 |  |
|  | *In bed alone* | 37.0 | 28.4 |  | 28.2 |  | 28.4 |  |
|  | *In bed near parent* | 13.7 | 13.3 |  | 11.7 |  | 12.9 |  |
| Night sleep duration | *Lowest quartile* | 20.1 | 23.8 | 0.46 | 35.9† | 0.006 | 27.0 | 0.09 |
|  | *2^nd^ quartile* | 23.7 | 26.2 |  | 22.3 |  | 25.2 |  |
|  | *3^rd^ quartile* | 27.9 | 27.3 |  | 27.2 |  | 27.3 |  |
|  | *Highest quartile* | 28.3 | 22.7 |  | 14.6 |  | 20.6 |  |
| Night wakings frequency | *0* | 19.6 | 13.6 | 0.33 | 12.6 | 0.49 | 13.4 | 0.23 |
| (times per night) | *1* | 29.7 | 30.8 |  | 32.0 |  | 31.1 |  |
|  | *2* | 30.1 | 32.2 |  | 32.0 |  | 32.1 |  |
|  | *3+* | 20.6 | 23.4 |  | 23.3 |  | 23.4 |  |

P-values for variables with multiple categories (e.g. maternal education) are comparing the overall distribution between the two relevant groups

TABLE E3. Food-specific versus overall EIG adherence status

|  | | | **Overall EIG per-protocol adherence status** | | |  | | |
| --- | --- | --- | --- | --- | --- | --- | --- | --- |
|  | | | **Adherence Evaluable (AE)** | | **Adherence Unknown** |  | | |
|  |  |  | **Adhered**  **(N=223)*** | **Nonadhered**  **(N=306)**† | **Nonevaluable**  **(N=123)**‡ | **Total**  **(N=652)** | | |
|  |  |  |  |  |  | **n** | **% (AE)** | **% (Total)** |
| **Food**  **Specific**  **EIG**  **Adherence**  **Status** | **Milk** | Adhered | 223 | 217 | 11 | 451 | 84.0 | 69.2 |
|  |  | Nonadhered | 0 | 80 | 6 | 86 | 16.0 | 13.2 |
|  |  | Nonevaluable | 0 | 9 | 106 | 115 |  | 17.6 |
|  | **Egg** | Adhered | 188 | 41 | 5 | 234 | 42.5 | 35.9 |
|  |  | Nonadhered | 35 | 262 | 20 | 317 | 57.5 | 48.6 |
|  |  | Nonevaluable | 0 | 3 | 98 | 101 |  | 15.5 |
|  | **Fish** | Adhered | 217 | 95 | 6 | 318 | 58.6 | 48.8 |
|  |  | Nonadhered | 6 | 205 | 14 | 225 | 41.4 | 34.5 |
|  |  | Nonevaluable | 0 | 6 | 103 | 109 |  | 16.7 |
|  | **Sesame** | Adhered | 214 | 72 | 2 | 288 | 52.4 | 44.2 |
|  |  | Nonadhered | 9 | 233 | 20 | 262 | 47.6 | 40.2 |
|  |  | Nonevaluable | 0 | 1 | 101 | 102 |  | 15.6 |
|  | **Peanut** | Adhered | 220 | 110 | 6 | 336 | 61.2 | 51.5 |
|  |  | Nonadhered | 3 | 195 | 15 | 213 | 38.8 | 32.7 |
|  |  | Nonevaluable | 0 | 1 | 102 | 103 |  | 15.8 |
|  | **Wheat** | Adhered | 184 | 31 | 1 | 216 | 39.1 | 33.1 |
|  |  | Nonadhered | 39 | 274 | 24 | 337 | 60.9 | 51.7 |
|  |  | Nonevaluable | 0 | 1 | 98 | 99 |  | 15.2 |

* EIG overall adhered: 223/529 (42.2% of adherence evaluable EIG participants), 223/652 (34.2% of total EIG)

† EIG overall nonadhered: 306/529 (57.8% of adherence evaluable EIG participants), 306/652 (46.9% of total EIG)

‡ EIG overall adherence non-evaluable: 123/652 (18.9% of total EIG)

AE Adherence Evaluable

TABLE E4. Enrollment factors influencing likelihood of being overall and food-specific nonadherent in the EIG (univariate analysis)

|  |  | **EIG Nonadherence**  Unadjusted odds ratio for being nonadherent compared with adherent | | | | | | |
| --- | --- | --- | --- | --- | --- | --- | --- | --- |
|  |  | **Overall** | **Food-specific** | | | | | |
|  |  |  | **Peanut** | **Egg** | **Milk** | **Sesame** | **Fish** | **Wheat** |
| **Demography** |  |  |  |  |  |  |  |  |
| Sex (female) |  | 1.89 | 1.15 | 1.24 | 1.27 | 1.13 | 0.84 | 1.06 |
| Ethnicity | *White (baseline)* | 1.00 | 1.00 | 1.00 | 1.00 | 1.00 | 1.00 | 1.00 |
|  | *Mixed* | 1.70 | 1.52 | 1.44 | 1.34 | 1.34 | 1.90 | 1.39 |
|  | *Black/Asian/Chinese* | 4.69† | 3.90† | 3.36† | 2.16 | 3.34† | 3.34† | 3.01* |
| Siblings (any) (%) |  | 1.22 | 1.11 | 1.26 | 1.47 | 1.27 | 1.37 | 1.10 |
| Pet ownership (any) |  | 0.78 | 0.75 | 0.76 | 0.70 | 0.83 | 0.63* | 0.72 |
| Maternal education | *>18 years (baseline)* | 1.00 | 1.00 | 1.00 | 1.00 | 1.00 | 1.00 | 1.00 |
|  | *17-18 years* | 1.00 | 0.93 | 0.85 | 0.59 | 1.16 | 0.81 | 0.91 |
|  | *16 years* | 1.39 | 1.25 | 1.22 | 1.39 | 1.13 | 0.72 | 1.06 |
| Paternal education | *>18 years* | 1.00 | 1.00 | 1.00 | 1.00 | 1.00 | 1.00 | 1.00 |
|  | *17-18 years* | 0.89 | 0.92 | 0.91 | 0.80 | 0.94 | 0.84 | 0.80 |
|  | *16 years* | 0.97 | 0.79 | 0.90 | 0.99 | 1.25 | 0.86 | 1.01 |
| **Smoking** |  |  |  |  |  |  |  |  |
| Maternal smoking |  | 0.73 | 0.79 | 0.74 | 0.73 | 0.65 | 1.80 | 1.01 |
| Paternal smoking |  | 0.91 | 1.11 | 0.62 | 0.73 | 1.15 | 0.91 | 0.96 |
| **Birth history** |  |  |  |  |  |  |  |  |
| Caesarean delivery |  | 1.26 | 1.16 | 1.10 | 1.40 | 1.18 | 1.41 | 1.22 |
| **Enrollment growth status** |  |  |  |  |  |  |  |  |
| Birth to enrollment weight gain | *Lowest quartile* | 1.00 | 1.00 | 1.00 | 1.00 | 1.00 | 1.00 | 1.00 |
|  | *2^nd^ quartile* | 1.13 | 1.90* | 1.27 | 2.16* | 1.16 | 1.65* | 1.39 |
|  | *3^rd^ quartile* | 1.27 | 1.58 | 1.45 | 1.83 | 1.18 | 1.94† | 1.32 |
|  | *Highest quartile* | 1.41 | 1.47 | 1.54 | 1.21 | 1.16 | 1.92† | 1.23 |
| Enrollment weight | *Lowest quartile* | 1.00 | 1.00 | 1.00 | 1.00 | 1.00 | 1.00 | 1.00 |
|  | *2^nd^ quartile* | 0.88 | 1.03 | 1.35 | 1.09 | 1.05 | 1.24 | 1.25 |
|  | *3^rd^ quartile* | 0.93 | 1.13 | 1.32 | 0.95 | 0.99 | 1.12 | 1.33 |
|  | *Highest quartile* | 1.38 | 1.27 | 1.56 | 1.32 | 1.32 | 1.55 | 1.32 |
| **Enrollment eczema** |  |  |  |  |  |  |  |  |
| Any visible eczema |  | 1.47 | 1.30 | 1.68* | 1.75* | 1.58* | 1.72* | 1.04 |
| SCORAD (continuous) |  | 1.03* | 1.03* | 1.04* | 1.03* | 1.04* | 1.04* | 1.01 |
| SCORAD severity at 3m visit | *None* | 1.00 | 1.00 | 1.00 | 1.00 | 1.00 | 1.00 | 1.00 |
|  | *SCORAD 1-15* | 1.38 | 1.16 | 1.68* | 1.77* | 1.30 | 1.57* | 0.93 |
|  | *SCORAD >15* | 1.92 | 1.93 | 1.68 | 0.68 | 3.15† | 2.44* | 1.59 |
| Raised TEWL |  | 1.22 | 1.42 | 1.36 | 0.97 | 1.37 | 1.31 | 0.99 |
| **Enrollment sensitization** |  |  |  |  |  |  |  |  |
| Skin-prick positive | Any food | 0.98 | 1.76 | 1.91 | 3.85† | 1.86 | 1.82 | 1.12 |
|  | Specific food | - | 8.09 | 1.76 | 8.53* | ** | ** | ** |
| Specific IgE positive (≥0.1 kU/l) | Any food | 1.16 | 1.22 | 1.40 | 1.30 | 1.09 | 1.65* | 1.05 |
|  | Specific food | - | 2.81* | 2.58* | 2.26 | 1.36 | 2.34 | 0.66 |
| **Family atopy status** |  |  |  |  |  |  |  |  |
| Maternal atopy |  | 1.25 | 0.91 | 1.25 | 1.08 | 1.05 | 0.96 | 1.07 |
| Paternal atopy |  | 0.98 | 0.98 | 0.98 | 1.00 | 0.84 | 0.96 | 1.00 |
| **Maternal factors** |  |  |  |  |  |  |  |  |
| Physical QOL | *Highest quartile* | 1.00 | 1.00 | 1.00 | 1.00 | 1.00 | 1.00 | 1.00 |
|  | *2^nd^ quartile* | 2.20† | 2.77‡ | 2.06† | 2.75† | 2.01† | 1.79* | 2.32† |
|  | *3^rd^ quartile* | 1.67* | 1.66 | 1.44 | 1.03 | 1.25 | 1.04 | 1.38 |
|  | *Lowest quartile* | 1.62 | 1.81 | 1.36 | 1.45 | 1.17 | 1.22 | 1.27 |
| Psychological QOL | *Highest quartile* | 1.00 | 1.00 | 1.00 | 1.00 | 1.00 | 1.00 | 1.00 |
|  | *2^nd^ quartile* | 0.77 | 0.70 | 0.92 | 0.70 | 1.14 | 1.06 | 0.59 |
|  | *3^rd^ quartile* | 2.07† | 2.01† | 1.72* | 1.39 | 1.66* | 1.63* | 2.07† |
|  | *Lowest quartile* | 1.70* | 1.53 | 1.30 | 1.10 | 1.22 | 1.22 | 1.37 |
| Social QOL | *Highest quartile* | 1.00 | 1.00 | 1.00 | 1.00 | 1.00 | 1.00 | 1.00 |
|  | *2^nd^ quartile* | 1.41 | 1.25 | 1.66 | 1.19 | 1.22 | 0.98 | 0.99 |
|  | *3^rd^ quartile* | 1.19 | 1.64 | 1.37* | 2.13* | 1.26 | 1.07 | 1.01 |
|  | *Lowest quartile* | 1.60 | 1.88* | 1.98† | 1.37 | 1.34 | 1.25 | 1.30 |
| Environment QOL | *Highest quartile* | 1.00 | 1.00 | 1.00 | 1.00 | 1.00 | 1.00 | 1.00 |
|  | *2^nd^ quartile* | 1.61 | 1.30 | 1.21 | 1.39 | 1.82* | 1.02 | 1.91* |
|  | *3^rd^ quartile* | 1.84* | 1.82* | 1.23 | 1.67 | 1.85* | 1.36 | 2.36‡ |
|  | *Lowest quartile* | 1.72* | 1.43 | 1.47 | 1.01 | 1.54 | 1.26 | 1.76* |
| Maternal age (33+) |  | 1.59* | 1.78† | 2.27‡ | 1.72* | 1.40 | 1.45* | 1.21 |
| **Paternal factors** |  |  |  |  |  |  |  |  |
| Paternal age (35+) |  | 1.43* | 1.40 | 1.53* | 1.36 | 1.36 | 1.49* | 1.16 |
| **Childcare** |  |  |  |  |  |  |  |  |
| Nursery/childcare |  | 1.19 | 1.37 | 0.86 | 0.45 | 1.78 | 2.30 | 1.03 |
| **Infant sleep at enrollment** |  |  |  |  |  |  |  |  |
| Where does baby sleep | *Cot in parents’ room* | 1.00 | 1.00 | 1.00 | 1.00 | 1.00 | 1.00 | 1.00 |
|  | *Cot in separate room* | 0.74 | 0.68 | 0.71 | 0.89 | 0.90 | 0.79 | 0.84 |
|  | *In parents’ bed* | 1.59 | 2.40† | 1.52 | 1.40 | 1.84* | 1.95* | 1.71 |
|  | *Cot in sibling’s room* | 0.30 | 0.28 | 0.29 | 1.00 | 0.48 | 0.61 | 0.52 |
| How baby falls asleep | *While feeding* | 1.00 | 1.00 | 1.00 | 1.00 | 1.00 | 1.00 | 1.00 |
|  | *Being rocked* | 0.94 | 1.05 | 0.81 | 0.52 | 0.56 | 0.92 | 1.07 |
|  | *Being held* | 0.76 | 0.75 | 0.80 | 1.00 | 0.55 | 0.65 | 0.85 |
|  | *In bed alone* | 0.62* | 0.56† | 0.73 | 0.62 | 0.59* | 0.61* | 0.63* |
|  | *In bed near parent* | 0.80 | 0.74 | 0.81 | 0.62 | 0.61 | 0.50* | 0.91 |
| Night sleep duration | *Lowest quartile* | 1.00 | 1.00 | 1.00 | 1.00 | 1.00 | 1.00 | 1.00 |
|  | *2^nd^ quartile* | 0.95 | 0.96 | 0.94 | 0.86 | 0.86 | 0.64 | 0.82 |
|  | *3^rd^ quartile* | 0.81 | 0.77 | 0.93 | 0.78 | 0.88 | 0.91 | 0.70 |
|  | *Highest quartile* | 0.69 | 0.61 | 0.62 | 0.76 | 0.82 | 0.63 | 0.73 |
| Night wakings frequency | *0* | 1.00 | 1.00 | 1.00 | 1.00 | 1.00 | 1.00 | 1.00 |
|  | *1* | 1.53 | 2.08* | 1.44 | 1.01 | 1.25 | 1.16 | 1.18 |
|  | *2* | 1.54 | 2.26† | 1.78* | 1.06 | 1.50 | 1.22 | 1.56 |
|  | *3+* | 1.66 | 1.89* | 1.67 | 1.09 | 1.44 | 1.41 | 1.42 |
| Parent reported sleep problem | *No problem* | 1.00 | 1.00 | 1.00 | 1.00 | 1.00 | 1.00 | 1.00 |
|  | *Small problem* | 1.21 | 1.41 | 1.00 | 1.27 | 1.10 | 1.20 | 1.12 |
|  | *Very serious problem* | 0.39 | 0.92 | - | - | 0.23 | 0.32 | 1.37 |

*p<0.05 †p<0.01 ‡p<0.001 **no infant sensitized in this category or predicts nonadherence perfectly and odds ratio can therefore not be calculated

TABLE E5. Association of ethnicity with demographic variables in the EAT study

|  | **White**  **(N=1104)** | **Mixed**  **(N=119)** | **Asian, Black  or Chinese**  **(N=80)** | **Between race**  **p value** |
| --- | --- | --- | --- | --- |
| **Demographic** |  |  |  |  |
| Sex (male) (%) | 50.5 | 47.9 | 48.8 | 0.84 |
| Siblings (any) (%) | 61.7 | 58.8 | 75.0 | 0.04 |
| Maternal education (≤18 years) (%) | 19.3 | 17.7 | 15.0 | 0.60 |
| **Smoking** |  |  |  |  |
| Maternal smoking (%) | 3.2 | 5.9 | 0.0 | 0.07 |
| Father smoking (%) | 10.2 | 17.7 | 8.9 | 0.04 |
| **Birth history** |  |  |  |  |
| Birth weight (mean kg) | 3.58 | 3.43 | 3.33 |  |
| *p value vs white* |  | 0.005 | <0.0005 |  |
| Caesarean delivery (%) | 24.3 | 30.3 | 30.0 | 0.21 |
| **Participant enrollment atopy status** |  |  |  |  |
| Visible eczema at 3m visit (%) | 22.6 | 30.3 | 39.2 | 0.001 |
| Scorad at 3m visit (median)* | 7.25 | 7.35 | 15.2 |  |
| *p value vs white* |  | 0.40 | <0.0005 |  |
| Any sensitisation at 3m visit (%) | 3.6 | 4.2 | 23.4 | <0.0005 |
| **Family atopy status** |  |  |  |  |
| Maternal asthma (%) | 27.3 | 26.1 | 12.7 | 0.02 |
| Maternal eczema (%) | 34.9 | 38.7 | 22.8 | 0.06 |
| Maternal atopy (%) | 63.6 | 62.2 | 49.4 | 0.04 |
| Paternal atopy (%) | 54.3 | 51.3 | 39.2 | 0.03 |
| **Family history of food allergy** |  |  |  |  |
| Sibling (%) | 16.8 | 17.7 | 29.1 | 0.02 |
| Mother (%) | 19.3 | 22.7 | 15.2 | 0.42 |
| Father (%) | 10.8 | 12.6 | 5.1 | 0.21 |
| **Maternal factors** |  |  |  |  |
| Maternal QOL at 3m mean (SD) |  |  |  |  |
| Physical | 16.6 (1.9) | 16.1 (1.9) | 15.4 (2.4) |  |
| *p value vs white* |  | 0.004 | <0.0005 |  |
| Psychological | 15.6 (2.0) | 15.4 (1.7) | 15.5 (2.5) |  |
| *p value vs white* |  | 0.22 | 0.58 |  |
| Social | 15.7 (2.7) | 15.0 (3.0) | 15.0 (3.0) |  |
| *p value vs white* |  | 0.01 | 0.06 |  |
| Environment | 16.5 (1.9) | 15.7 (1.9) | 15.3 (2.1) |  |
| *p value vs white* |  | 0.0001 | <0.0005 |  |
| Maternal age (mean years) | 33 | 34 | 33 |  |
| *p value vs white* |  | 0.12 | 0.14 |  |
| **Participation measures** |  |  |  |  |
| Number of IQ completed  (median - max 17) | 16 | 16 | 11.5 |  |
| *p value vs white* |  | <0.0005 | <0.0005 |  |
| **Breastfeeding data** |  |  |  |  |
| Duration of any breastfeeding  (mean weeks) | 50 | 52 | 52 |  |
| *p value vs white* |  | 0.13 | 0.85 |  |
| Duration of exclusive breastfeeding  (mean weeks) | 18 | 21 | 18 |  |
| *p value vs white* |  | 0.009 | 0.81 |  |

***TABLE E6. Post enrollment factors through to six months influencing likelihood of being overall and food-specific nonadherent in the EIG (univariate analysis)***

|  |  | **EIG Nonadherence**  Unadjusted odds ratio for being nonadherent compared with adherent | | | | | | |
| --- | --- | --- | --- | --- | --- | --- | --- | --- |
|  |  |  | **Food-specific** | | | | | |
|  |  | **Overall** | **Peanut** | **Egg** | **Milk** | **Sesame** | **Fish** | **Wheat** |
| **Eczema natural history** |  |  |  |  |  |  |  |  |
| New onset eczema (4-6m) |  | 1.29 | 1.26 | 0.96 | 0.79 | 1.07 | 0.99 | 1.02 |
| **Parent reported symptoms with food consumption (4-6m)** |  |  |  |  |  |  |  |  |
| (A) IgE-type symptoms | (1) To one or more EI foods | 2.26‡ | 1.39 | 3.23‡ | 1.56 | 2.32‡ | 1.49 | 1.67* |
|  | (2) To any other food | 0.92 | 1.33 | 1.12 | 3.25 | 1.10 | 0.94 | 0.64 |
|  | (1 or 2) To any food | 2.14† | 1.39 | 3.03‡ | 1.73* | 2.22‡ | 1.43 | 1.46 |
| (B) Non-IgE-type symptoms | (1) To one or more EI foods | 1.50 | 1.42 | 1.55 | 1.46 | 1.34 | 1.32 | 1.07 |
|  | (2) To any other food | 0.88 | 1.14 | 0.52 | 0.49 | 0.78 | 1.01 | 1.13 |
|  | (1 or 2) To any food | 1.28 | 1.30 | 1.44 | 1.39 | 1.25 | 1.20 | 0.99 |
| (A or B) Any food symptoms | (1) To one or more EI foods | 2.01† | 1.51* | 2.58‡ | 1.42 | 1.95† | 1.48* | 1.46 |
|  | (2) To any other food | 1.02 | 1.20 | 0.73 | 1.31 | 0.90 | 0.94 | 0.96 |
|  | (1 or 2) To any food | 1.78† | 1.40 | 2.38‡ | 1.48 | 1.80† | 1.33 | 1.28 |
| **Food-specific symptoms (4-6m)** |  |  |  |  |  |  |  |  |
|  | IgE-type symptoms | - | 5.40† | 9.06‡ | 3.03† | 5.11† | 5.28 | 2.38 |
|  | Non-IgE-type symptoms | - | 1.30 | 1.85 | 3.03† | 1.10 | 3.33 | 0.64 |
|  | Any food symptoms | - | 3.09† | 4.76‡ | 2.52† | 2.68* | 3.16 | 1.39 |
| **Feeding difficulties/aversion (4m)** |  |  |  |  |  |  |  |  |
| Any difficulty feeding baby | *No difficulty (baseline)* | 1.00 | 1.00 | 1.00 | 1.00 | 1.00 | 1.00 | 1.00 |
| what you want them to eat? | *Occasional difficulty* | 0.86 | 0.76 | 1.12 | 0.50 | 0.55* | 0.76 | 0.68 |
|  | *Some difficulty* | 2.12† | 2.09† | 1.77* | 2.10* | 1.84* | 2.05† | 1.50 |
|  | *Great difficulty* | 17.69‡ | 5.19‡ | 5.53‡ | 9.41‡ | 7.00‡ | 7.30‡ | 9.10‡ |
| Refuses food? | *Never (baseline)* | 1.00 | 1.00 | 1.00 | 1.00 | 1.00 | 1.00 | 1.00 |
|  | *Rarely* | 1.02 | 1.04 | 1.04 | 0.74 | 0.92 | 0.81 | 0.72 |
|  | *Sometimes* | 1.38 | 1.73* | 1.41 | 0.76 | 1.19 | 1.47 | 1.11 |
|  | *Frequently* | 4.19* | 4.01† | 2.81* | 3.86† | 6.57† | 5.04† | 3.62* |
|  | *Always* | - | 8.64† | - | 15.5‡ | 13.14† | 7.56* | 6.51 |
| Turns head away when | *Never* | 1.00 | 1.00 | 1.00 | 1.00 | 1.00 | 1.00 | 1.00 |
| offered food? | *Rarely* | 1.01 | 0.81 | 1.26 | 0.77 | 0.69 | 0.94 | 0.90 |
|  | *Sometimes* | 1.90* | 1.84* | 1.61 | 1.08 | 1.88* | 1.53 | 1.25 |
|  | *Frequently* | 3.77† | 4.17† | 2.79* | 4.53† | 3.57† | 5.32‡ | 3.80† |
|  | *Always* | ** | 6.94 | ** | ** | ** | 8.11 | 3.16 |
| Spits food out? | *Never* | 1.00 | 1.00 | 1.00 | 1.00 | 1.00 | 1.00 | 1.00 |
|  | *Rarely* | 0.64 | 0.55 | 0.82 | 0.77 | 0.68 | 0.49 | 0.51* |
|  | *Sometimes* | 1.21 | 0.84 | 1.67* | 0.90 | 0.75 | 1.03 | 0.60 |
|  | *Frequently* | 1.76 | 1.18 | 2.29† | 2.39* | 1.47 | 1.13 | 1.15 |
|  | *Always* | 7.21* | 2.85* | 22.4† | 8.18‡ | 4.63* | 6.56† | 3.23 |
| Baby shuts mouth when | *Never* | 1.00 | 1.00 | 1.00 | 1.00 | 1.00 | 1.00 | 1.00 |
| offered food? | *Rarely* | 0.76 | 0.72 | 0.98 | 0.47 | 0.62* | 0.71 | 0.68 |
|  | *Sometimes* | 1.44 | 1.46 | 1.68* | 1.00 | 0.95 | 1.12 | 1.36 |
|  | *Frequently* | 1.87 | 1.98 | 1.83 | 2.39 | 1.41 | 1.79 | 1.53 |
|  | *Always* | ** | 5.27 | ** | 17.20† | 8.14 | 11.73* | ** |

*p<0.05 †p<0.01 ‡p<0.001 **These cells predict nonadherence perfectly hence no odds ratio can be calculated

**TABLE E7. Feeding difficulties and food refusal by EIG per-protocol status and primary outcome status - 4 months of age**

|  |  | **EIG Per-Protocol status** | | | |  |
| --- | --- | --- | --- | --- | --- | --- |
|  |  | **Per-Protocol**  **(A)**  (N=223) | **Non-Per-Protocol**  **(B)**  (N=306) | **Adherence Nonevaluable**  **(C)**  (N=123) | **Non-Per-Protocol or Adherence Nonevaluable**  **(B+C)**  (N=429) | **EIG Primary**  **Outcome**  **Positive**  **(%)** |
| **Any difficulty feeding baby** |  | (N=201) | (N=237) | (N=37) | (N=274) |  |
| **what you want them to eat?** | *No difficulty* | 41.8 | 34.2 | 37.8 | 34.7 | 6.2 |
|  | *Occasional difficulty* | 38.3 | 26.2 | 32.4 | 27.0 | 4.6 |
|  | *Some difficulty* | 18.9 | 28.3 | 21.6 | 27.4 | 6.2 |
|  | *Great difficulty* | 1.0 | 11.4 | 8.1 | 11.0 | 6.3 |
|  | p value |  | (AvB)  <0.0005 | (AvC)  0.04 | (Av(B+C))  <0.0005 | 0.93 |
| **Refuses food?** |  | (N=200) | (N=232) | (N=37) | (N=269) |  |
|  | *Never* | 47.5 | 42.2 | 54.1 | 43.9 | 4.7 |
|  | *Rarely* | 35.5 | 30.6 | 24.3 | 29.7 | 6.0 |
|  | *Sometimes* | 15.5 | 17.7 | 13.5 | 17.1 | 7.8 |
|  | *Frequently* | 1.5 | 6.5 | 8.1 | 6.7 | 4.8 |
|  | *Always* | 0.0 | 3.0 | 0.0 | 2.6 | 14.3 |
|  | p value |  | (AvB)  0.007 | (AvC)  0.08 | (Av(B+C))  0.008 | 0.73 |
| **Turns head away when** |  | (N=200) | (N=231) | (N=37) | (N=274) |  |
| **offered food?** | *Never* | 52.5 | 44.2 | 48.7 | 44.8 | 4.0 |
|  | *Rarely* | 31.5 | 26.4 | 18.9 | 25.4 | 6.9 |
|  | *Sometimes* | 13.0 | 19.5 | 29.7 | 20.9 | 8.5 |
|  | *Frequently* | 3.0 | 8.7 | 2.7 | 7.8 | 3.7 |
|  | *Always* | 0.0 | 1.3 | 0.0 | 1.1 | 33.3 |
|  | p value |  | (AvB)  0.009 | (AvC)  0.06 | (Av(B+C))  0.008 | 0.13 |
| **Spits food out?** |  | (N=200) | (N=231) | (N=37) | (N=274) |  |
|  | *Never* | 22.5 | 20.4 | 21.6 | 20.6 | 4.0 |
|  | *Rarely* | 24.5 | 13.6 | 21.6 | 14.7 | 4.5 |
|  | *Sometimes* | 34.5 | 35.3 | 32.4 | 34.9 | 5.5 |
|  | *Frequently* | 17.5 | 25.5 | 24.3 | 25.4 | 8.7 |
|  | *Always* | 1.0 | 5.1 | 0.0 | 4.4 | 7.1 |
|  | p value |  | (AvB)  0.003 | (AvC)  0.86 | (Av(B+C))  0.008 | 0.64 |
| **Baby shuts mouth when** |  | (N=200) | (N=231) | (N=37) | (N=274) |  |
| **offered food?** | *Never* | 42.2 | 40.1 | 46.0 | 40.9 | 4.1 |
|  | *Rarely* | 34.7 | 24.6 | 24.3 | 24.5 | 3.0 |
|  | *Sometimes* | 18.6 | 24.6 | 27.0 | 24.9 | 11.5 |
|  | *Frequently* | 4.5 | 8.6 | 2.7 | 7.8 | 10.0 |
|  | *Always* | 0.0 | 2.2 | 0.0 | 1.9 | 0.0 |
|  | p value |  | (AvB)  0.01 | (AvC)  0.48 | (Av(B+C))  0.02 | 0.03 |

**TABLE E8. Number of EIG families reporting food-specific non-IgE and IgE-type symptoms in the key early introduction period in the EIG**

|  |  | **Food-specific symptoms** | | | | | | **Any food symptoms^†^** |
| --- | --- | --- | --- | --- | --- | --- | --- | --- |
|  |  | **Peanut** | **Egg** | **Milk** | **Sesame** | **Fish** | **Wheat** |
| **4 months** | *IgE-type* | 3 | 3 | 10 | 2 | 0 | 0 | 15 |
|  | *Non-IgE-type* | 4 | 5 | 20 | 3 | 1 | 0 | 27 |
|  | *IgE and/or non-IgE-type* | 5 | 7 | 25 | 5 | 1 | 0* | 36 |
| **5 months** | *IgE-type* | 8 | 39 | 21 | 12 | 3 | 5 | 63 |
|  | *Non-IgE-type* | 3 | 22 | 28 | 7 | 2 | 6 | 52 |
|  | *IgE and/or non-IgE-type* | 10 | 52 | 39 | 16 | 3 | 9 | 98 |
| **6 months** | *IgE-type* | 13 | 20 | 8 | 7 | 3 | 1 | 41 |
|  | *Non-IgE-type* | 3 | 8 | 8 | 1 | 1 | 0 | 19 |
|  | *IgE and/or non-IgE-type* | 14 | 26 | 16 | 7 | 3 | 1 | 55 |
| **4-6 months** | *IgE-type* | 22 | 52 | 33 | 18 | 6 | 6 | 99 |
|  | *Non-IgE-type* | 10 | 28 | 46 | 11 | 4 | 6 | 82 |
|  | *IgE and/or non-IgE-type* | 27 | 68 | 63 | 25 | 7 | 10 | 149 |

Symptoms were divided into IgE-type (urticaria, angioedema, acute rash, respiratory symptoms, acute vomiting) or non-IgE-type symptoms (diarrhoea, constipation, reflux, delayed vomiting, delayed rash, eczema)

*Wheat not introduced before 4 months of age in EIG

†Symptoms to one or more of the six early introduction allergenic foods
