# Supplementary material for: Factors influencing adherence in a trial of early introduction of allergenic food
Source: J Allergy Clin Immunol. 2019 Dec;144(6):1595–605. doi: 10.1016/j.jaci.2019.06.046 (PMC6904906; doi:10.1016/j.jaci.2019.06.046)
Supplement: Legends for Figs E1-E9 [file mmc2.docx]

**Supplementary Appendix Figure Legends**

**FIG E1.Likely true adherence status of the EIG adherence nonevaluable participants**

For each of the six early introduction foods, in the left hand panels, the percentage of the weekly recommended dose of allergenic protein consumed from enrollment to 12 months of age is presented by *overall adherence status*. The EIG group is divided into those who were overall adherent (N=223; blue line), those who were overall nonadherent (N=306; red line), and those in whom overall adherence was nonevaluable (N=123; green line).

The middle panels show the percentage of the weekly recommended dose of allergenic protein consumed by *food-specific adherence status*. Food-specific adherence varied considerably by food and significantly exceeded overall adherence for a number of foods (Table E3). The number of EIG participants who were adherent (blue), nonadherent (red) and adherence nonevaluable (green) for each specific food is shown in the respective middle panel.

In the right hand panels, instead of looking at the weekly dose being consumed, as in the left and central panels, the percentage of EIG infants who were consuming a specific food at the per-protocol threshold (75% or more of the recommended dose) is presented.

**FIG E2. The association between EIG overall adherence status and the weekly total number of allergenic foods being consumed at per-protocol level and weekly achievement of consumption of five or more foods at 75% or more of the recommended dose in the key early introduction period (to six months of age)**

Panel A shows the number of foods being consumed at or above the per-protocol threshold at any particular time point by overall adherence status. Panel B shows the percentage of EIG participants who were meeting or exceeding the per-protocol definition of five or more foods being consumed at 75% or more of the recommended dose by overall adherence status. In Panel C, the interim questionnaire completion rate is shown for the 123 EIG participants whose overall adherence status was nonevaluable.

FIG E3. Order of introduction of the four randomised foods and overall and food-specific per-protocol adherence

The order of introduction of four of the six allergenic foods (peanut, egg, fish and sesame) was determined randomly. The effect of the position in the order in which each of these four foods was introduced on food-specific per-protocol compliance and overall per-protocol compliance is presented.

FIG E4. Effect of ethnicity, maternal age, eczema SCORAD severity group and parent reported feeding difficulties at 4 months of age on the weekly total number of allergenic foods being consumed at per-protocol level by the EIG and weekly achievement of consumption of five or more foods at 75% or more of the recommended dose.

The left hand panels show the number of foods being consumed at or above the per-protocol threshold at any particular time point by: Panel A Ethnicity; Panel B Maternal age; Panel C Enrollment eczema severity; and Panel D Family reported infant feeding difficulty at 4 months of age. The right hand panels show the percentage of EIG participants who were meeting or exceeding the per-protocol definition of five or more foods being consumed at 75% or more of the recommended dose. This figure excludes the seven enrollment challenge positive infants (1 wheat, 4 milk, 2 egg and 2 peanut) who were instructed to avoid consumption of that food completely.

**FIG E5. EIG enrollment IgE sensitization and the development of food allergy**

Penalized logistic regression of the association between enrollment IgE sensitization (0.1 kU/l or greater) to specific foods, or to one or more of the six early introduction foods, and the development of food allergy to specific foods, or to one or more of the six early introduction foods (primary outcome).

**FIG E6. Consumption of each allergenic food by enrollment specific IgE sensitization status**

Each panel shows the percentage of the recommended weekly dose of each allergenic food consumed, by enrollment specific IgE sensitization (0.1 kU/l or greater) to that food. Numbers of EIG participants nonsensitized are in blue and sensitized in red. In brackets are the number of EIG participants who were sensitized on skin prick testing to the specific food (wheal ≥1mm).

This figure excludes the seven enrollment challenge positive infants (1 wheat, 4 milk, 2 egg and 2 peanut) who were instructed to avoid consumption of that food completely.

FIG E7. Parent reporting of IgE and non-IgE type symptoms to early introduction foods, or any other food, by adherence status

The percentage of participants reporting any symptoms (left hand panels), IgE type symptoms (central panels) and non-IgE type symptoms (right hand panels) with food consumption is shown for each month from enrollment to 12 months of age. Top row is for symptoms with one or more of the six early introduction foods, middle row is for symptoms with any other food and bottom row is for symptoms with any food. Data are presented for the adherent SIG participants (blue line), adherent EIG participants (red line) and nonadherent EIG participants (green line).

**FIG E8. The reporting in the key early introduction period (up to six months) of IgE-type symptoms to specific foods, IgE or non-IgE-type symptoms to any of the early introduction foods, and the association with food-specific and overall food allergy**

Penalized logistic regression of the association between parent reported symptoms with consumption of the six allergenic foods reported by 6 months of age and the development of food allergy to one or more foods (primary outcome), or to specific foods. Symptoms manifesting by six months of age are presented for IgE-type symptoms for each specific food, IgE-type symptoms to one or more of the six early introduction foods and non-IgE-type symptoms to one or more of the six early introduction foods.

FIG E9. Interrelationship between ethnicity, enrollment IgE sensitization and enrollment visible eczema in both EAT study groups and their effects on per-protocol adherence and the development of food allergy

The Venn diagram examines how the combination of risk factors for allergy and per-protocol adherence are related. Rectangular areas represent the total number of participants in the respective ethnic group. All areas, rectangles and Venn diagrams, are to scale with regards to the number of participants. Within each region of the diagram, the sample size and percentage of the respective ethnic group is indicated on the top row. The second row displays the per-protocol adherence rate in the EIG and SIG participants respectively in the specific region. The third row displays the primary outcome of food allergy prevalence in the EIG and SIG participants respectively in the specific region.
