# Supplementary figures and images for: Factors influencing adherence in a trial of early introduction of allergenic food
Source: J Allergy Clin Immunol. 2019 Dec;144(6):1595–605. doi: 10.1016/j.jaci.2019.06.046 (PMC6904906; doi:10.1016/j.jaci.2019.06.046)

Milk

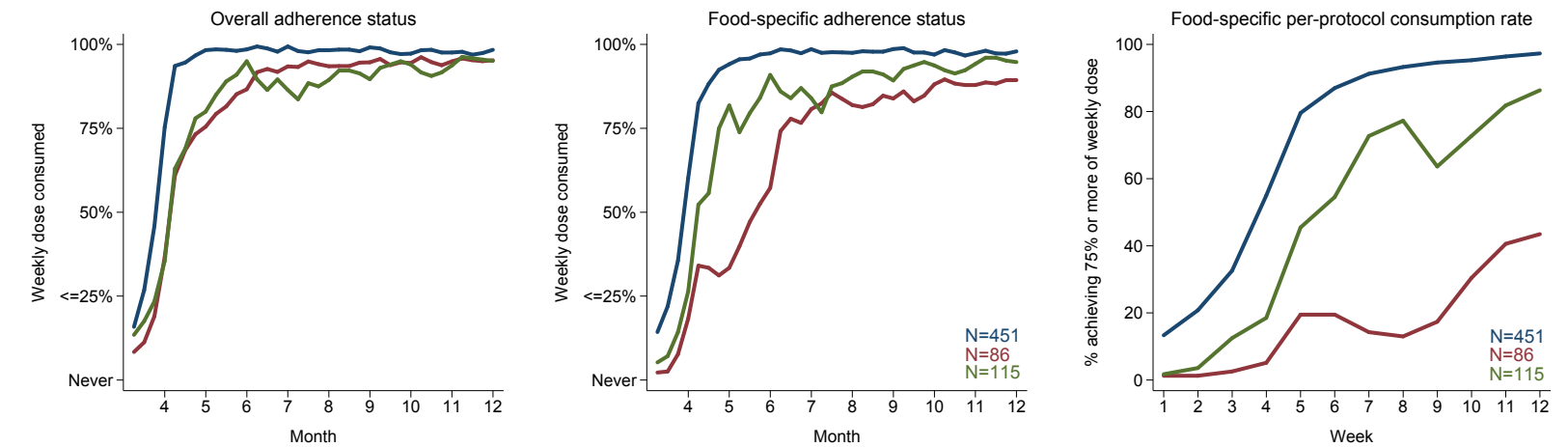

Egg

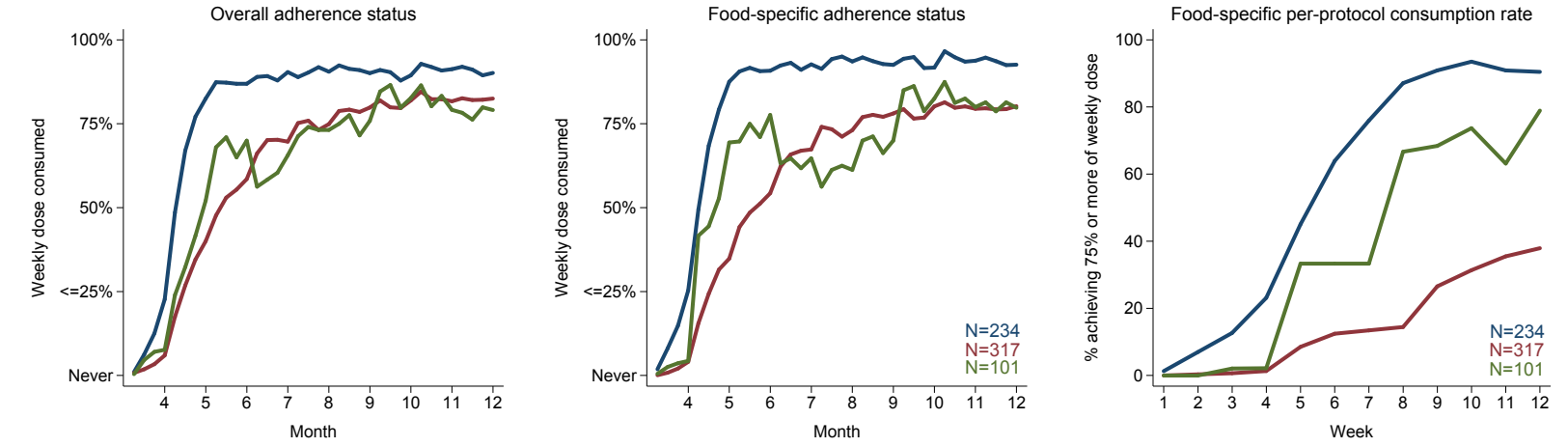

Fish

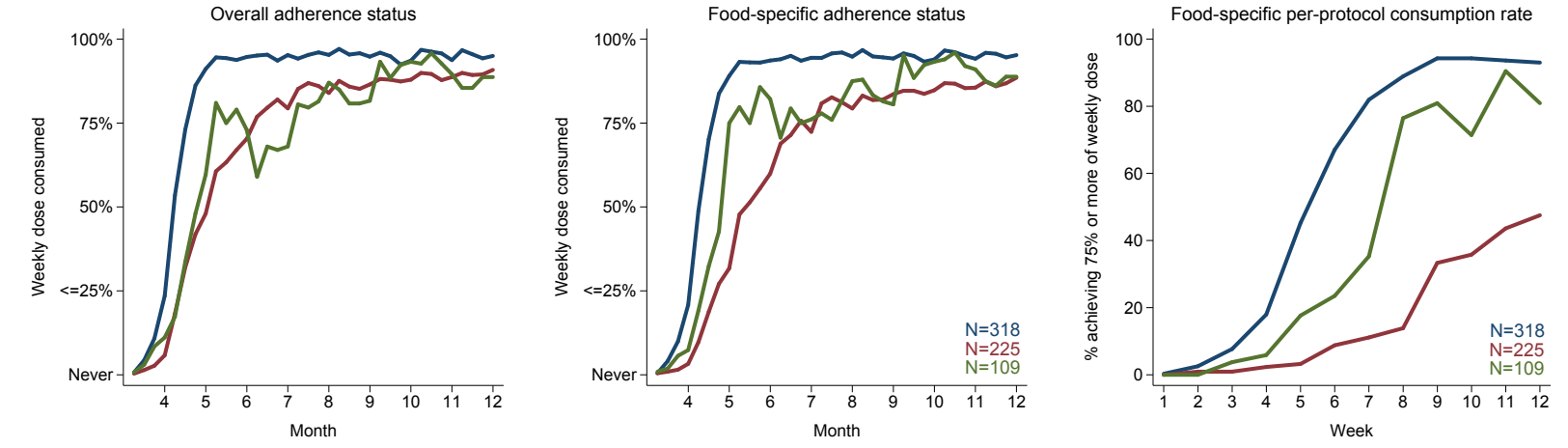

Sesame

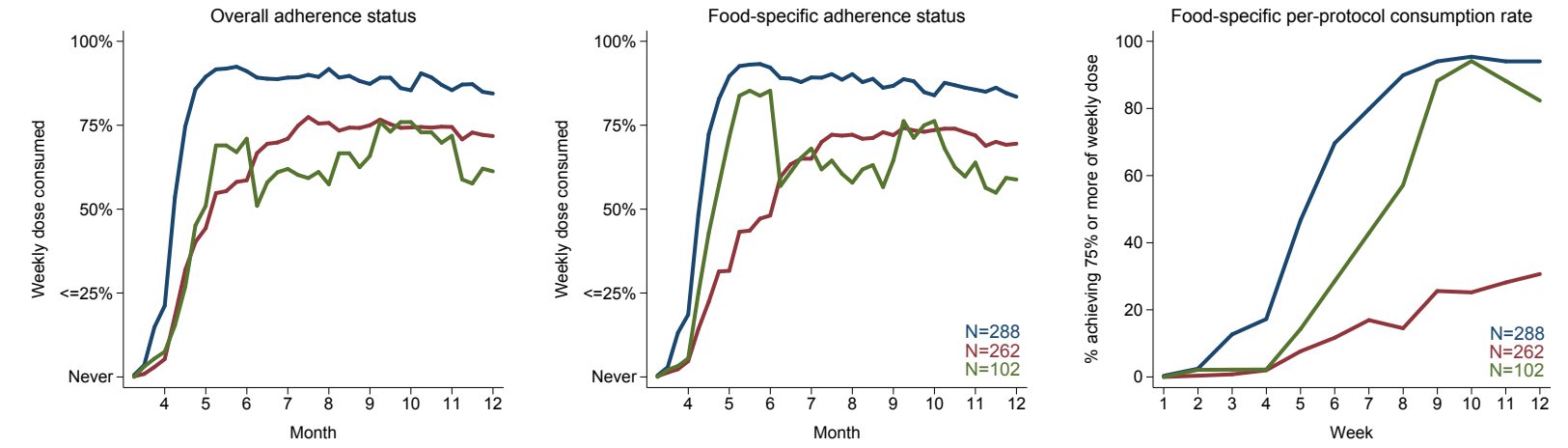

Peanut

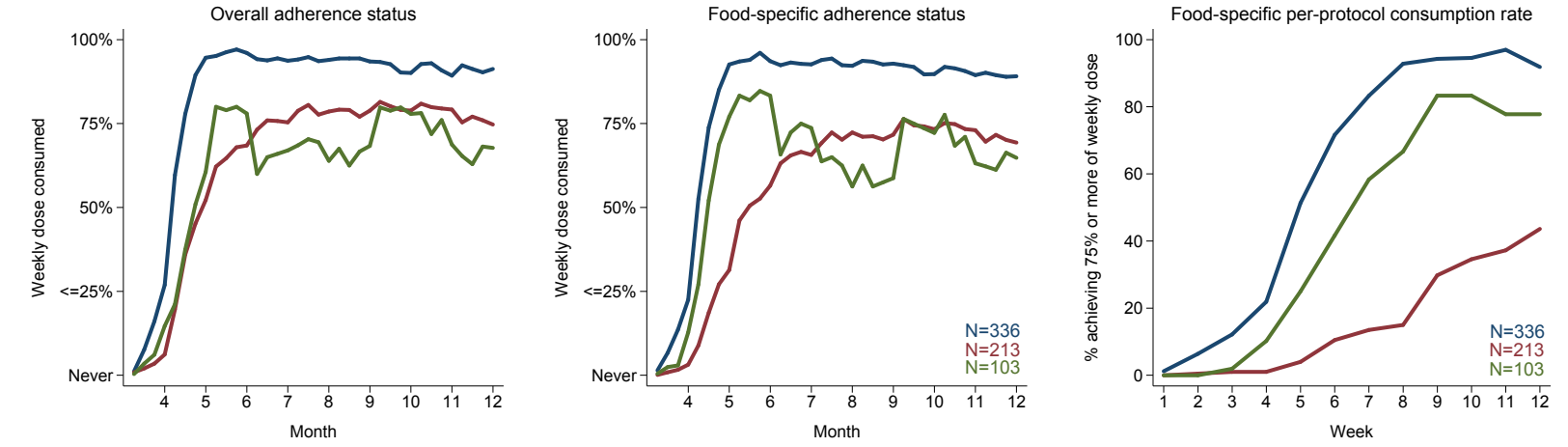

Wheat

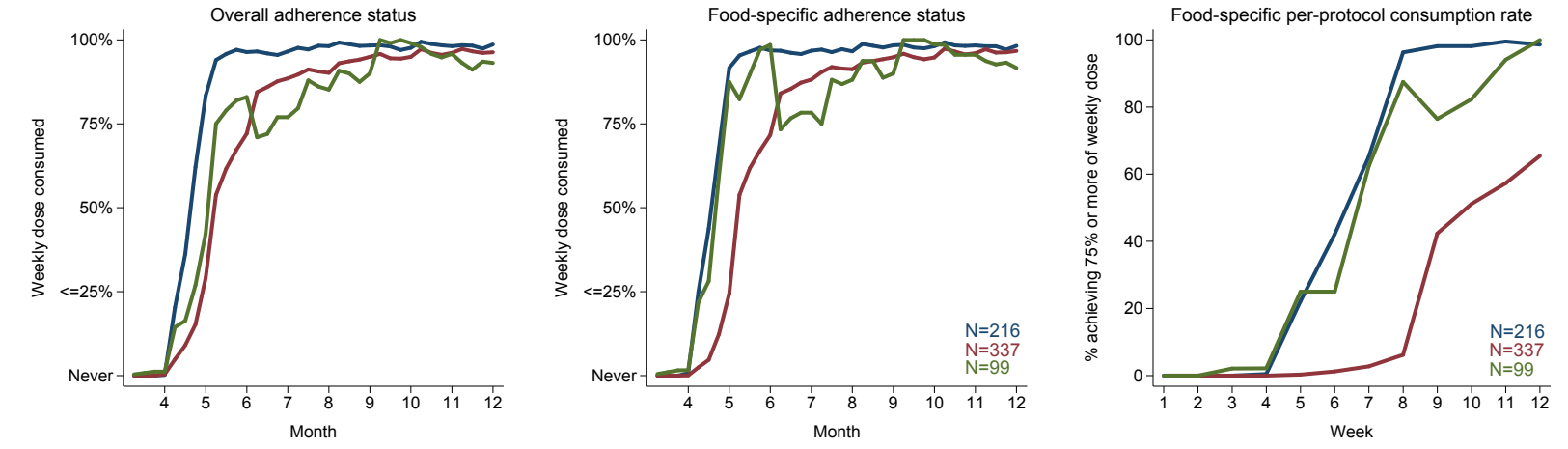

Complied

Noncomplied

Nonevaluable

Supplement: Fig E1 [file mmc3.pdf]

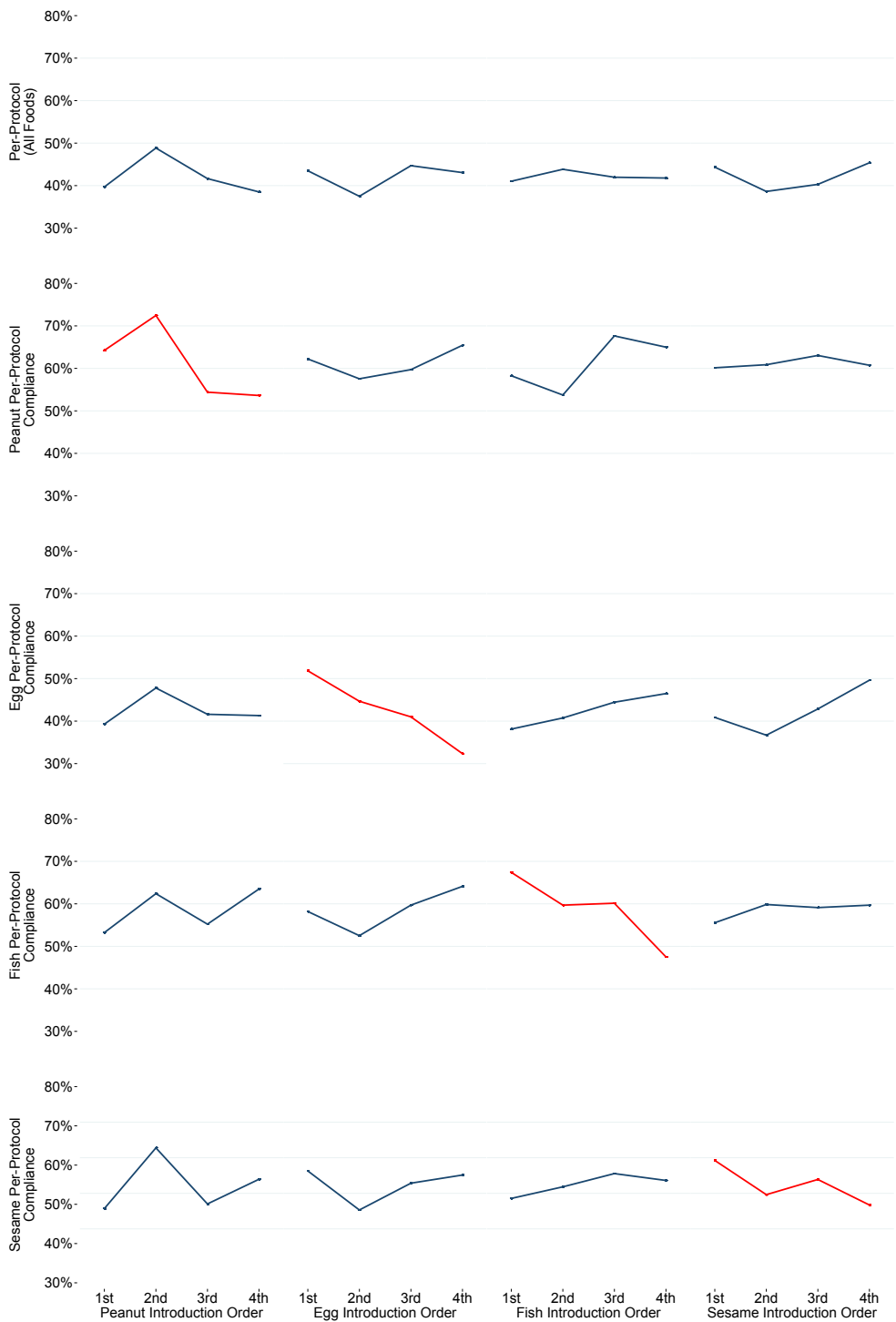

Supplement: Fig E3 [file mmc5.pdf]

A: Ethnicity

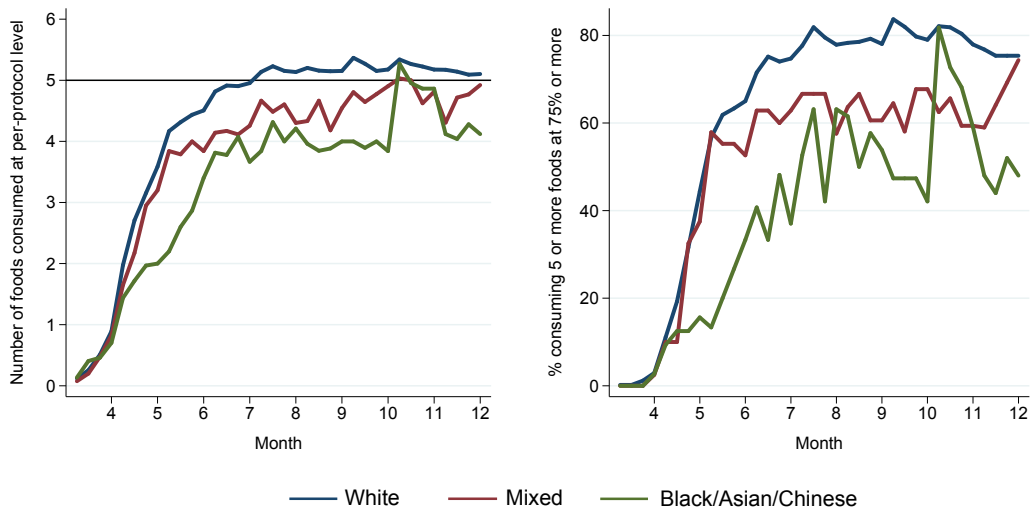

B: Maternal age

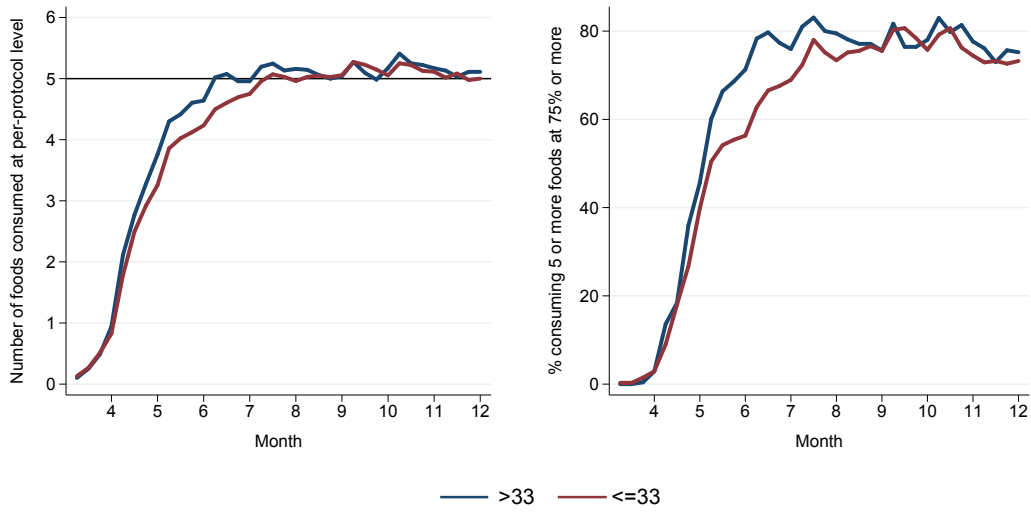

C: Eczema severity

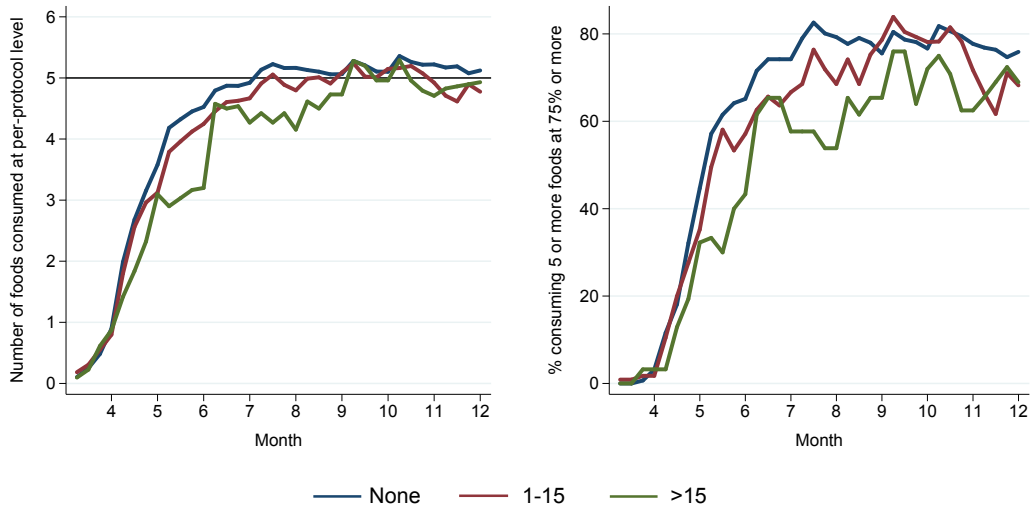

D: Feeding difficulty 4m

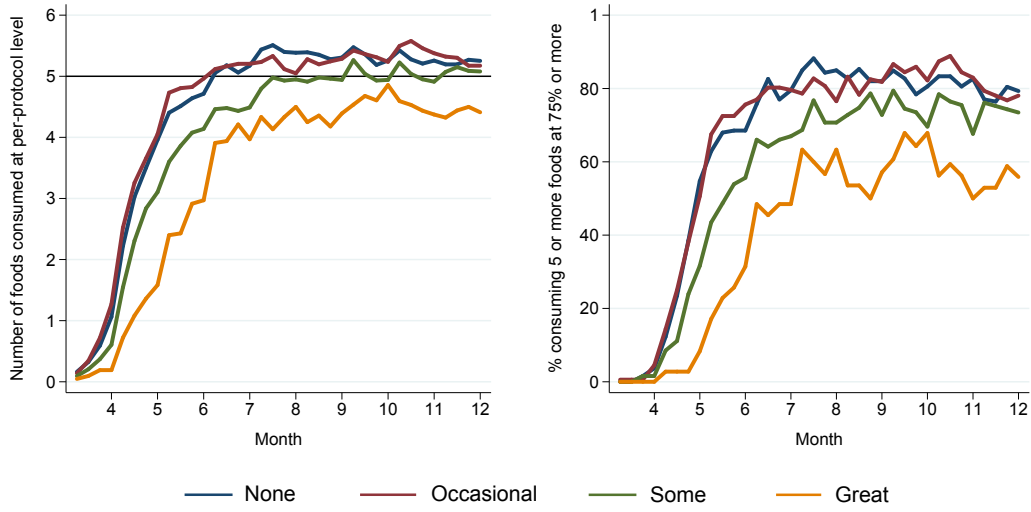

Supplement: Fig E4 [file mmc6.pdf]

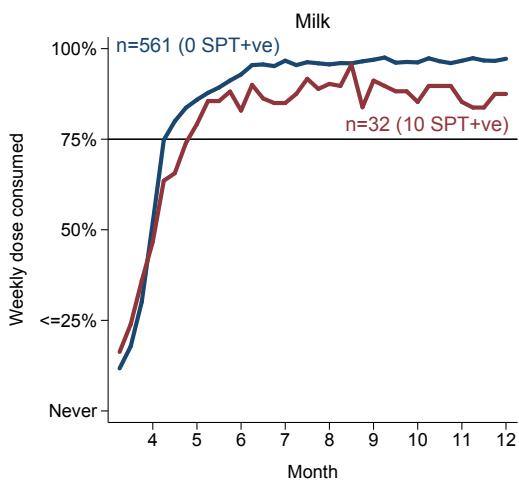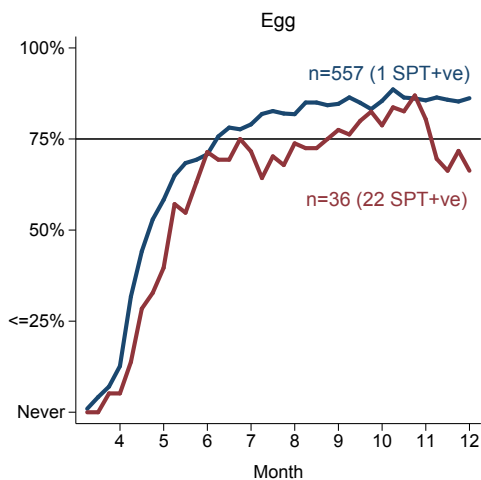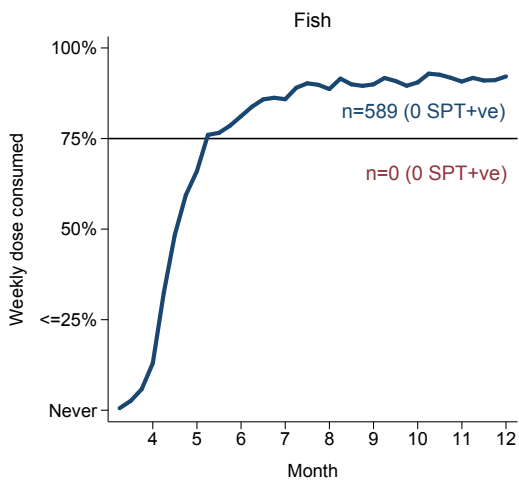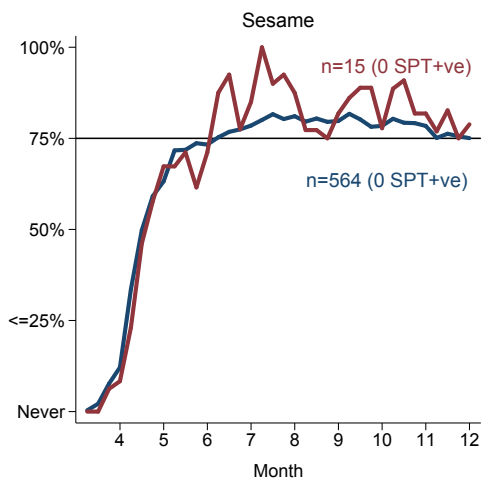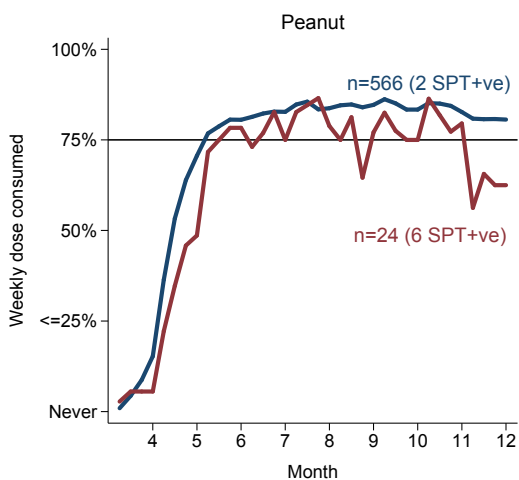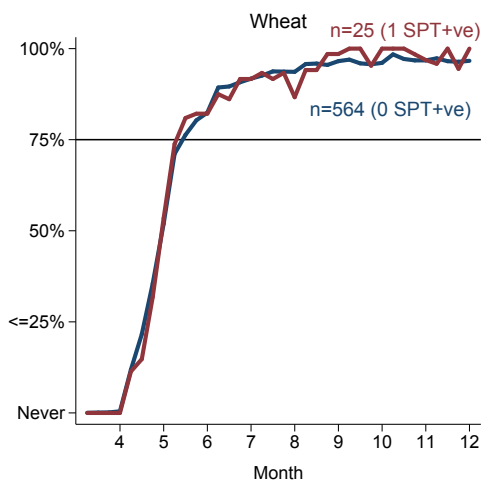

— Not sensitized — Sensitized (0.1 kU/l or greater)

Supplement: Fig E6 [file mmc8.pdf]
