# Supplementary material for: Factors influencing adherence in a trial of early introduction of allergenic food
Source: J Allergy Clin Immunol. 2019 Dec;144(6):1595–605. doi: 10.1016/j.jaci.2019.06.046 (PMC6904906; doi:10.1016/j.jaci.2019.06.046)
Supplement: Fig E2 [file mmc4.pdf]

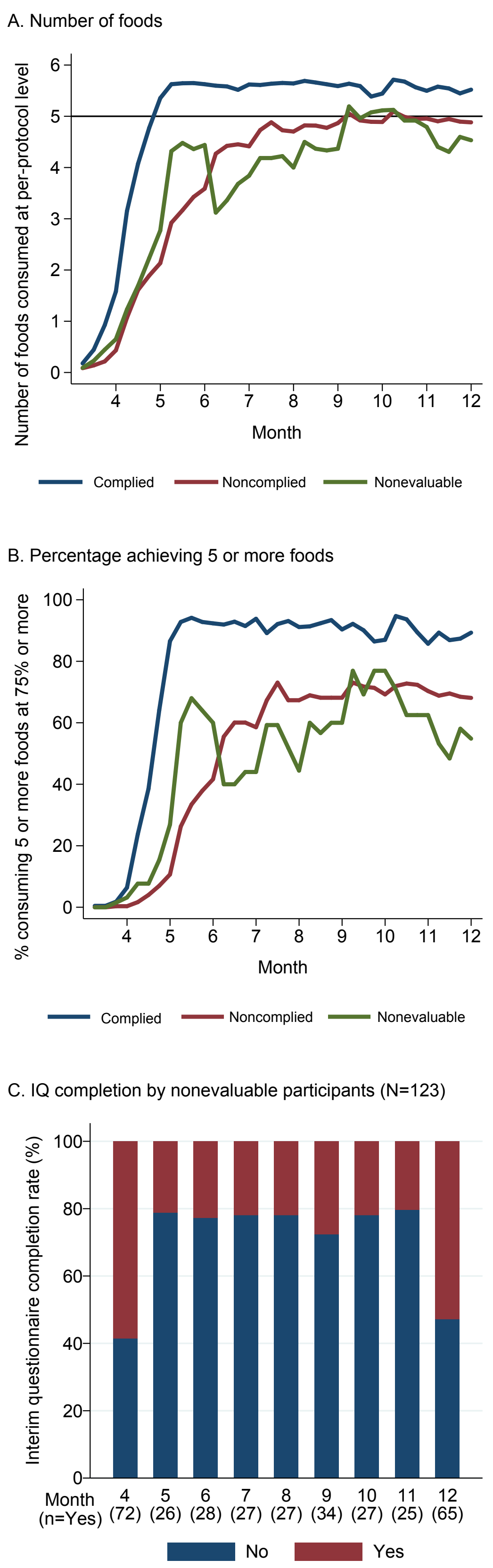
A. Number of foods
Number of foods consumed at per-protocol level
0
1
2
3
4
5
6
4 5 6 7 8 9 10 11 12
Month
Complied
Noncomplied
Nonevaluable
B. Percentage achieving 5 or more foods
% consuming 5 or more foods at 75% or more
0
20
40
60
80
100
4 5 6 7 8 9 10 11 12
Month
Complied
Noncomplied
Nonevaluable
C. IQ completion by nonevaluable participants (N=123)
Interim questionnaire completion rate (%)
0
20
40
60
80
100
Month (n=Yes)
4 (72)
5 (26)
6 (28)
7 (27)
8 (27)
9 (34)
10 (27)
11 (25)
12 (65)
No
Yes
