# Supplementary material for: Factors influencing adherence in a trial of early introduction of allergenic food
Source: J Allergy Clin Immunol. 2019 Dec;144(6):1595–605. doi: 10.1016/j.jaci.2019.06.046 (PMC6904906; doi:10.1016/j.jaci.2019.06.046)
Supplement: Fig E5 [file mmc7.pdf]

EIG allergy to individual foods	Peanut (n=24)	Egg (n=36)	Milk (n=32)	Sesame (n=15)	Fish (n=0)	Wheat (n=25)	One or more foods (n=93)
**Primary outcome Allergy to one or more foods (n=28)**	**7.33 (2.54-21.1) <0.001**	**7.62 (2.98-19.4) <0.001**	**16.2 (6.59-40.0) <0.001**	**4.17 (1.00-17.4) 0.05**	- - -	**4.01 (1.18-13.6) 0.03**	**8.35 (3.79-18.4) <0.001**
**Wheat (n=1)**	**72.8 (2.88-1842) 0.009**	**50.0 (1.99-1253) 0.02**	**53.8 (2.14-1352) 0.02**	13.3 (0.51-342) 0.12	- - -	**76.7 (3.03-1943) 0.009**	16.7 (0.67-413) 0.09
**Fish (n=0)**	23.2 (0.45-1196) 0.12	15.6 (0.3-799) 0.17	17.4 (0.34-890) 0.16	36.9 (0.71-1931) 0.07	- - -	24.4 (0.47-1259) 0.11	5.42 (0.11-275) 0.40
**Sesame (n=2)**	4.62 (0.22-99.2) 0.33	**16.1 (1.62-159) 0.02**	3.46 (0.16-73.7) 0.43	**39.8 (3.87-410) 0.002**	- - -	4.85 (0.23-104) 0.31	5.47 (0.56-53.3) 0.14
**Milk (n=2)**	**25.4 (2.52-255) 0.006**	3.56 (0.17-76.0) 0.42	**100.5 (4.70-2151) 0.003**	13.2 (0.51-340) 0.12	- - -	**80.5 (3.17-2044) 0.008**	**29.2 (1.39-615) 0.03**
**Egg (n=20)**	**5.86 (1.68-20.5) 0.006**	**9.36 (3.38-26.0) <0.001**	**14.1 (5.14-38.5) <0.001**	3.10 (0.53-17.9) 0.21	- - -	3.70 (0.91-15.1) 0.07	**8.54 (3.40-21.5) <0.001**
**Peanut (n=7)**	**20.7 (4.75-90.2) <0.001**	**7.47 (1.60-34.8) 0.01**	**15.5 (3.63-66.6) <0.001**	**9.06 (1.41-58.3) 0.02**	- - -	5.82 (0.93-36.3) 0.06	**7.35 (1.78-30.4) 0.006**

EIG Food-specific IgE sensitization at enrollment (0.1 kU/l or greater)
