# Supplementary material for: Factors influencing adherence in a trial of early introduction of allergenic food
Source: J Allergy Clin Immunol. 2019 Dec;144(6):1595–605. doi: 10.1016/j.jaci.2019.06.046 (PMC6904906; doi:10.1016/j.jaci.2019.06.046)
Supplement: Fig E7 [file mmc9.pdf]

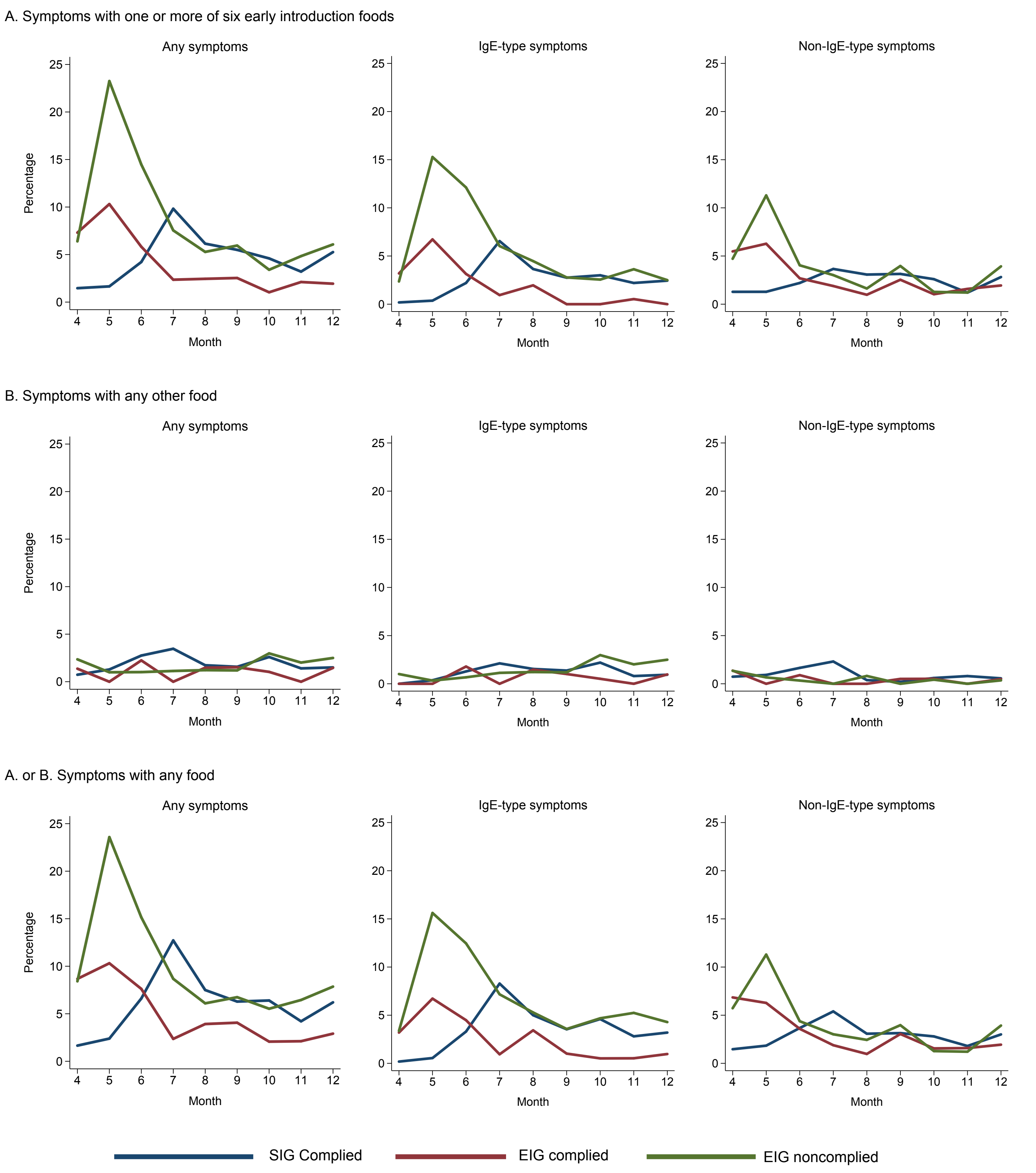
A. Symptoms with one or more of six early introduction foods
Any symptoms
IgE-type symptoms
Non-IgE-type symptoms
Percentage
Month
B. Symptoms with any other food
Any symptoms
IgE-type symptoms
Non-IgE-type symptoms
Percentage
Month
A. or B. Symptoms with any food
Any symptoms
IgE-type symptoms
Non-IgE-type symptoms
Percentage
Month
SIG Complied
EIG complied
EIG noncomplied
