# Supplementary material for: Factors influencing adherence in a trial of early introduction of allergenic food
Source: J Allergy Clin Immunol. 2019 Dec;144(6):1595–605. doi: 10.1016/j.jaci.2019.06.046 (PMC6904906; doi:10.1016/j.jaci.2019.06.046)
Supplement: Fig E8 [file mmc10.pdf]

EIG allergy to individual foods	Peanut (n=22)	Egg (n=52)	Milk (n=33)	Sesame (n=18)	Fish (n=6)	Wheat (n=6)	Any IgE-type symptoms (n=99)	Any non-IgE-type symptoms (n=82)
**Primary outcome Allergy to one or more foods (n=28)**	1.25<br>(0.23-6.83)<br>0.80	**7.34<br>(3.31-16.3)<br><0.001**	**5.09<br>(1.97-13.2)<br>0.001**	1.48<br>(0.27-8.21)<br>0.65	1.80<br>(0.09-34.1)<br>0.70	4.55<br>(0.72-28.7)<br>0.11	**7.01<br>(3.35-14.7)<br><0.001**	**6.71<br>(3.17-14.2)<br><0.001**
**Wheat (n=1)**	8.59<br>(0.34-217)<br>0.19	3.36<br>(0.14-83.7)<br>0.46	5.48<br>(0.22-137)<br>0.30	10.1<br>(0.40-257)<br>0.16	**40.3<br>(1.44-1130)<br>0.03**	**27.8<br>(1.03-749)<br>0.05**	1.60<br>(0.06-39.7)<br>0.77	2.23<br>(0.09-55.2)<br>0.63
**Fish (n=0)**	8.61<br>(0.34-217)<br>0.19	3.37<br>(0.14-83.8)<br>0.46	5.49<br>(0.22-137)<br>0.30	10.1<br>(0.40-258)<br>0.16	**40.4<br>(1.44-1131)<br>0.03**	**27.9<br>(1.04-750)<br>0.05**	1.61<br>(0.06-39.7<br>0.77	20.1<br>(0.81-498)<br>0.07
**Sesame (n=2)**	3.68<br>(0.18-73.5)<br>0.39	1.44<br>(0.07-28.3)<br>0.81	2.34<br>(0.12-46.3)<br>0.58	4.33<br>(0.22-87.1)<br>0.34	17.3<br>(0.77-385)<br>0.07	11.9<br>(0.56-254)<br>0.11	0.69<br>(0.04-13.4)<br>0.80	4.00<br>(0.52-30.7)<br>0.18
**Milk (n=2)**	3.65<br>(0.18-73.0)<br>0.40	1.49<br>(0.08-29.3)<br>0.79	**30.1<br>(3.85-236)<br>0.001**	**19.2<br>(2.39-154)<br>0.005**	17.1<br>(0.77-382)<br>0.07	11.8<br>(0.55-253)<br>0.11	**8.4<br>(1.09-64.0<br>0.04**	4.03<br>(0.52-31.0)<br>0.18
**Egg (n=20)**	2.00<br>(0.36-11.2)<br>0.43	**15.7<br>(6.13-40.2)<br><0.001**	**4.95<br>(1.63-15.0)<br>0.005**	0.71<br>(0.04-12.2)<br>0.81	2.83<br>(0.15-54.4)<br>0.49	**7.29<br>(1.13-46.9)<br>0.04**	**15.9<br>(5.83-43.2)<br><0.001**	**7.57<br>(3.09-18.5)<br><0.001**
**Peanut (n=7)**	6.18<br>(0.99-38.6)<br>0.05	2.40<br>(0.40-14.5)<br>0.34	4.01<br>(0.65-24.6)<br>0.13	2.00<br>(0.11-36.4)<br>0.64	7.96<br>(0.39-161)<br>0.18	5.49<br>(0.28-107)<br>0.26	2.24<br>(0.49-10.1)<br>0.30	3.14<br>(0.69-14.3)<br>0.14

Food-specific IgE-type symptoms reported up to 6 months
