# Supplementary material for: Factors influencing adherence in a trial of early introduction of allergenic food
Source: J Allergy Clin Immunol. 2019 Dec;144(6):1595–605. doi: 10.1016/j.jaci.2019.06.046 (PMC6904906; doi:10.1016/j.jaci.2019.06.046)
Supplement: Fig E9 [file mmc11.pdf]

White (n=816)

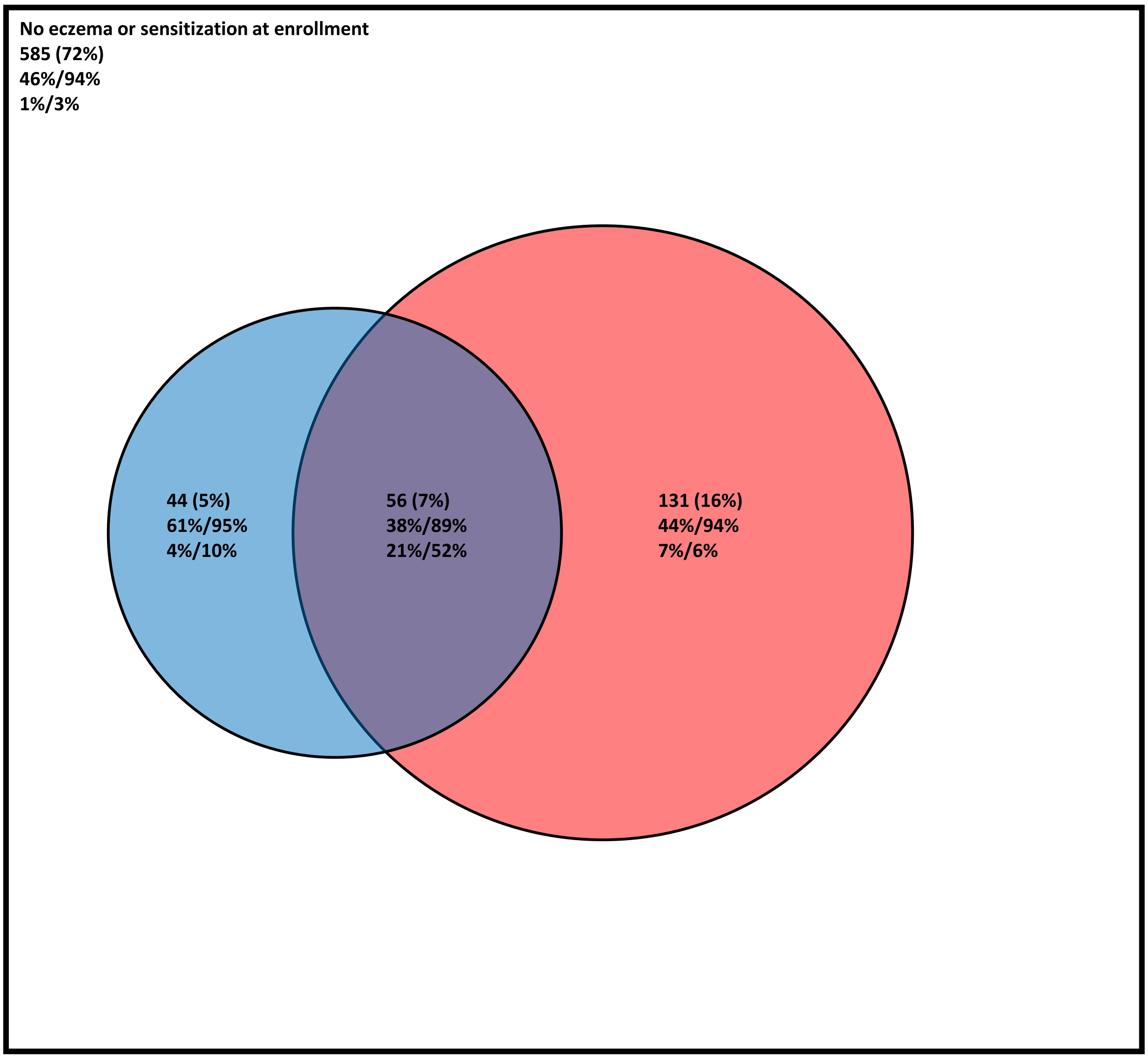

Nonwhite (n=126)

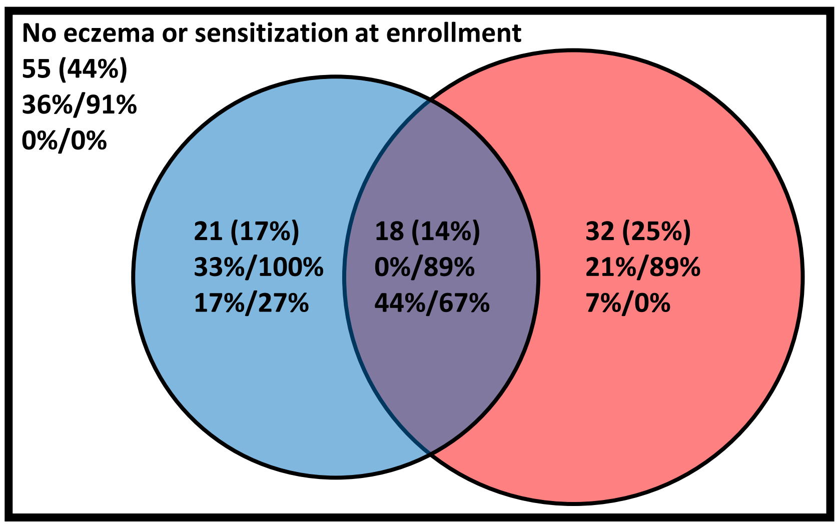

Key

IgE sensitized (≥0.1kU/l) one or more foods

Visible eczema at enrollment

N (%) in each region
Probability of EIG/SIG per-protocol adherence
Probabilty of food allergy EIG/SIG
